# Fecal microbiota transplantation for the decolonization of mucosal adherent-invasive *Escherichia coli* in Crohn’s disease: an open-label pilot study

**DOI:** 10.1038/s41467-026-77455-7

**Published:** 2026-09-10

**Authors:** Zhilu Xu, Hui Jiang, Wenli Huang, Alicia Chan, Yu Lin, Tingting Chan, Rashid N. Lui, Marc T. L. Wong, Joyce W. Y. Mak, Francis K. L. Chan, Caroline Chevarin, Jean-Frédéric Colombel, Anthony Buisson, Siew C. NG

**Affiliations:** 1 Microbiota I-Center (MagIC), Hong Kong SAR, China; 2 Department of Medicine and Therapeutics, State Key Laboratory of Digestive Disease, Institute of Digestive Disease, The Chinese University of Hong Kong, Hong Kong SAR, China; 3 Department of Psychiatry, The Chinese University of Hong Kong, Hong Kong SAR, China; 4 Department of Medicine and Therapeutics, Faculty of Medicine, The Chinese University of Hong Kong, Hong Kong SAR, China; 5 Center for Gut Microbiota Research, The Chinese University of Hong Kong, Hong Kong SAR, China; 6 Microbes, Intestin, Inflammation et Susceptibilité de l’Hôte (M2iSH), Université Clermont Auvergne/Inserm U1071, USC INRAE 1382, Clermont-Ferrand, France; 7 Department of Gastroenterology, Icahn School of Medicine, Mount Sinai, NY, USA; 8 Service d’Hépato-Gastro Entérologie, Université Clermont Auvergne, Inserm, 3iHP, CHU Clermont-Ferrand, Clermont-Ferrand, France; 9 New Cornerstone Science Laboratory, The Chinese University of Hong Kong, Hong Kong SAR, China

**Keywords:** Microbiome, Crohn's disease

## Abstract

Adherent-invasive *Escherichia coli* (AIEC) is implicated in Crohn’s disease (CD) pathogenesis, but whether fecal microbiota transplantation (FMT) clears mucosal AIEC and alters fecal and mucosal microbiomes has not been studied. In this open-label pilot study, thirty adults with CD and seropositivity for serum anti-*E.coli* antibody (AEcAb) received a single colonoscopic FMT from one of two donors (ClinicalTrials.gov identifier NCT05611866). The primary outcome was adverse events (AEs) within 12 weeks after FMT; secondary outcomes included changes in mucosal AIEC, clinical measures, and fecal and mucosal microbiome profiles. Shotgun metagenomic sequencing for fecal samples and 16S rRNA sequencing for ileal mucosal samples were performed at baseline and after FMT. Baseline AEcAb levels were higher in patients with mucosal AIEC colonization (*p* = 0.019) and remained elevated after FMT. No FMT-related serious adverse events were reported. FMT was associated with mucosal AIEC decolonization in all 8 evaluable baseline-positive patients. Mucosal alpha diversity increased after FMT (*p* = 0.023), which correlated with reduced fecal calprotectin (*p* = 0.034). Microbial pathway profiles in CD patients resembled those of their respective donors with reduced donor-recipient dissimilarity post-FMT (*p* = 0.030), particularly among recipients with mucosal AIEC decolonization (*p* = 0.039). In conclusion, this open-label pilot study demonstrates the feasibility of FMT for modulating mucosal AIEC colonization in CD and highlights the importance of capturing microbial changes at the disease site that may be undetectable in fecal samples.

## Abstract (web summary)

Here, in a pilot study in humans, the authors provide proof of concept that fecal microbiota transplantation can be used for clearing harmful *E. coli* from the gut lining of people with Crohn’s disease, highlighting the potential of microbiome-targeted therapies in inflammatory bowel disease.

## Introduction

Crohn’s disease (CD), one of the primary forms of inflammatory bowel disease (IBD), is a chronic inflammatory disorder of the gastrointestinal tract^1^. Its pathogenesis involves a complex interplay between environmental factors, genetic susceptibility, and intestinal dysbiosis, which collectively drive a dysregulated mucosal immune response^2–4^. The global incidence of CD is rising, a trend particularly evident in newly industrialized countries^5^, underscoring the need for improved therapeutic strategies. Accumulating evidence implicates specific pathobionts in CD pathogenesis^6^, with adherent-invasive *Escherichia coli* (AIEC) emerging as a key candidate^7,8^. Defined by its ability to adhere to and invade intestinal epithelial cells and survive within macrophages, AIEC is found at higher frequencies in the ileal mucosa of CD patients^9–12^ and was shown to induce and exacerbate inflammation in murine models^13,14^. This positions AIEC as a compelling therapeutic target. To date, no established therapy exists to selectively eradicate AIEC in patients with CD.

Fecal microbiota transplantation (FMT), a strategy to restore a healthy gut microbial ecosystem, presents a potential approach to eliminate pathobionts such as AIEC. While FMT has demonstrated efficacy in recurrent *Clostridioides difficile* infection^15^, its efficacy in CD has been modest and inconsistent^16–21^. This variability may partially stem from the lack of mechanistically-informed, microbiota-specific standards for donor selection beyond conventional pathogen screening^22^. This gap is particularly relevant in CD, where dysbiosis and pathobiont colonization may influence both microbiome remodeling and clinical response. Critically, the relationship between donor microbial engraftment, the clearance of specific pathobionts like AIEC, and clinical response in CD remains poorly understood.

To test this, we conducted an open-label pilot study to evaluate the safety, feasibility, and microbiological effects of FMT in patients with CD and seropositivity for anti-*E.coli* antibody (AEcAb). We assessed changes in mucosal AIEC colonization and characterized FMT-associated microbial shifts using longitudinal fecal and mucosal microbiome profiling. We found that a single FMT was associated with mucosal AIEC decolonization. This finding suggests the possibility that AIEC may represent a modifiable target in CD, warranting further investigation into its clinical relevance.

## Results

### Patient characteristics

**Fig. 1:**
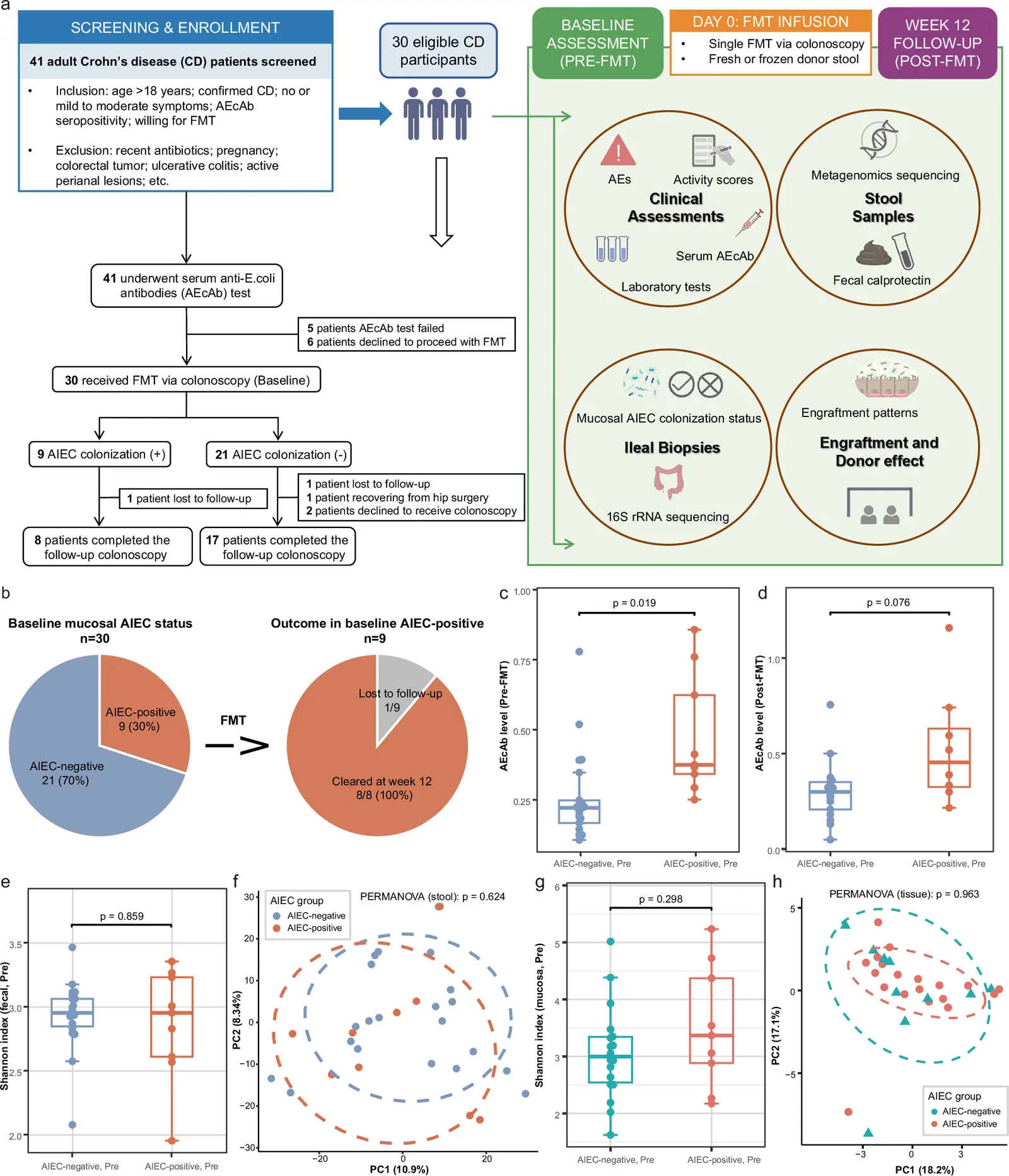
Study design, participant flow, and baseline stratification by mucosal AIEC status. **a** Study schema and participant flow. Adult CD patients were screened, enrolled, and received a single FMT via colonoscopy at baseline. Assessments at baseline and Week 12 included clinical and laboratory measures, fecal calprotectin, stool metagenomic sequencing, and ileal biopsies for mucosal AIEC status and 16S rRNA sequencing. Graphical elements: Created in BioRender. JIANG, H. (2026) https://BioRender.com/1r95k7r. **b** Baseline mucosal AIEC status and post-FMT outcome. Pi charts show baseline AIEC status (*n* = 30) and outcomes among baseline AIEC-positive recipients (*n* = 9), including loss to follow-up and AIEC clearance at Week 12. **c** Baseline serum AEcAb levels by mucosal AIEC status. AIEC-negative (*n* = 21) and AIEC-positive (*n* = 9) recipients were compared using a two-sided Welch’s t-test. **d** Week-12 serum AEcAb levels by baseline mucosal AIEC status. AIEC-negative (*n* = 17) and AIEC-positive (*n* = 8) recipients were compared using a two-sided Welch’s t-test. **e** Baseline fecal alpha diversity by mucosal AIEC status. AIEC-negative (*n* = 21) and AIEC-positive (*n* = 9) recipients were compared using a two-sided Wilcoxon rank-sum test. **f** Baseline fecal beta diversity by mucosal AIEC status. PCoA of baseline fecal microbiota based on Aitchison distance. Each point represents one baseline fecal sample (AIEC-negative, *n* = 21; AIEC-positive, *n* = 9); confidence ellipses are shown. Group differences were assessed using PERMANOVA. **g** Baseline mucosal alpha diversity by mucosal AIEC status. AIEC-negative (*n* = 18) and AIEC-positive (*n* = 9) recipients with available ileal biopsies were compared using a two-sided Wilcoxon rank-sum test. For **c**–**e** and **g**, box plots show the median (center line), 25th-75th percentiles (box), and the most extreme values within 1.5 times the interquartile range (whiskers); points represent individual participants. **h** Baseline mucosal beta diversity by mucosal AIEC status. PCoA of baseline mucosal microbiota based on Aitchison distance. Each point represents one baseline mucosal sample (AIEC-negative, *n* = 18; AIEC-positive, *n* = 9); confidence ellipses are shown. Group differences were assessed using PERMANOVA. CD Crohn’s disease; AEcAb anti-*E. coli* antibody; FMT fecal microbiota transplantation; AEs adverse events; AIEC adherent-invasive *E. coli*; PCoA principal coordinate analysis; PERMANOVA permutational multivariate analysis of variance.

Between October 2022 and January 2023, 41 adults with CD were screened, and 30 eligible patients received a single colonoscopic infusion of FMT in the terminal ileum (Fig. 1a). The mean age of participants was 35.6 years, and 8 (26.7%) were female (Table 1). Baseline fecal samples were available for all 30 participants, and baseline ileal biopsies were available for 27 participants. At week 12, 25 participants completed follow-up and provided fecal samples, and 24 provided ileal biopsies; paired mucosal samples were available in 22 participants.

**Table 1:** Patient baseline characteristics

Characteristics	All patients (*n* = 30)	Baseline AIEC colonization (+) (*n* = 9)	Baseline AIEC colonization (-) (*n* = 21)	*P* value
Age	35.6 (12.1)	33.4 (12.6)	36.5 (12.1)	0.551
Gender, Female, *n* (%)	8 (26.7%)	4 (44.4%)	4 (19.0%)	0.195
Disease location, *n* (%)				1.000
Ileal	2 (6.7%)	0	2 (9.5%)	/
Colonic	8 (26.7%)	2 (22.2%)	6 (28.6%)	/
Ileocolonic	20 (66.7%)	7 (77.8%)	13 (61.9%)	/
**Disease activity scores**				
HBI	0.9 (1.1)	0.8 (1.2)	1.0 (1.1)	0.712
CDAI	54.2 (37.1)	53.6 (33.8)	54.5 (39.2)	0.950
**Behavior phenotype,** ***n* (%)**				
Inflammatory	29 (96.7%)	8 (88.9%)	21 (100%)	0.300
Stricturing	4 (13.3%)	2 (22.2%)	2 (9.5%)	0.563
Penetrating / fistulising	3 (10.0%)	2 (22.2%)	1 (4.8%)	0.207
Perianal, *n* (%)	2 (6.7%)	2 (22.2%)	0	0.083
**Medication,** ***n* (%)**				
Topical Treatment	3 (10.0%)	2 (22.2%)	1 (4.8%)	0.207
Systematic 5-Aminosalicylic Acid	8 (26.7%)	2 (22.2%)	6 (28.6%)	1.000
Systematic Steroids	0	0	0	/
Immunosuppressants	20 (66.7%)	4 (44.4%)	16 (76.2%)	0.115
Anti-Tumor Necrosis Factor	15 (50.0%)	5 (55.6%)	10 (47.6%)	1.000
Antibiotics	1 (3.3%)	1 (11.1%)	0	0.300
**Inflammatory markers**				
Fecal Calprotectin (µg/g)	679.6 (799.6)	960.6 (853.1)	559.2 (764.9)	0.244
CRP (mg/L)	2.7 (4.9)	3.6 (6.5)	2.4 (4.2)	0.608
ESR (mm/h)	17.7 (14.0)	16.9 (8.7)	18.0 (15.9)	0.808
**Hematology markers**				
Hb (g/dL)	13.9 (1.5)	13.1 (1.4)	14.2 (1.4)	0.066
RBC (× 10^12^/L)	4.8 (0.7)	4.7 (0.8)	4.8 (0.7)	0.632
WBC (× 10^9^/L)	6.5 (1.6)	6.9 (1.9)	6.4 (1.5)	0.496
PLT (× 10^9^/L)	297.4 (64.2)	310.7 (59.6)	291.7 (66.6)	0.451
**Biochemical indicators**				
Albumin (g/L)	40.1 (4.0)	38.3 (5.2)	40.9 (3.2)	0.208
ALP (U/L)	73.6 (24.8)	71.0 (17.3)	74.8 (27.7)	0.657
ALT (U/L)	24.0 (14.9)	20.4 (10.9)	25.5 (16.4)	0.335
TB (µmol/L)	10.2 (5.7)	12.2 (8.4)	9.3 (4.1)	0.339
Creatinine (µmol/L)	74.4 (12.2)	70.1 (10.0)	76.2 (12.9)	0.179
Potassium (mmol/L)	4.0 (0.3)	3.8 (0.3)	4.0 (0.3)	0.198
Sodium (mmol/L)	140.4 (1.7)	139.8 (1.9)	140.7 (1.7)	0.235

Abbreviations: *AIEC* Adherent-Invasive *E. coli*; *HBI* Harvey-Bradshaw Index; *CDAI* Crohn’s Disease Activity Index; *CRP* C-Reactive Protein; *ESR* Erythrocyte Sedimentation Rate; *Hb* Hemoglobin; *RBC* Red Blood Cell count; *WBC* White Blood Cell count; *PLT* Platelets; *ALP* Alkaline Phosphatase; *ALT* Alanine Aminotransferase; *TB* Total Bilirubin.

Continuous variables were expressed as mean ± standard deviation (SD) and compared using two-sided Welch’s t-test. Categorical variables were presented as counts and percentages, and compared using two-sided Fisher’s exact test.

At baseline, 9/30 participants (30%) had mucosal AIEC colonization (Fig. 1b). Baseline characteristics were comparable between AIEC-positive and AIEC-negative participants (Table 1). Serum AEcAb levels were significantly higher in AIEC-positive participants compared with AIEC-negative participants (0.48 ± 0.22 vs 0.26 ± 0.16; *P* = 0.019; Fig. 1c). At week 12, AEcAb levels were not significantly different between baseline AIEC-positive participants and baseline AIEC-negative participants (*P* = 0.076; Fig. 1d). Fecal and mucosal microbiome diversity and composition were comparable between AIEC-positive and AIEC-negative participants at baseline. There were no differences in fecal Shannon diversity (*P* = 0.397), fecal Aitchison-based beta diversity (PERMANOVA, *P* = 0.624), mucosal Shannon diversity (*P* = 0.298), or mucosal beta diversity (PERMANOVA, *P* = 0.963) between AIEC-positive and AIEC-negative participants at baseline (Fig. 1e–h).

#### FMT was well tolerated and associated with AIEC clearance in patients with CD

Two patients experienced adverse events: one developed mild diarrhea six days after FMT, and another patient had gastrointestinal bleeding 44 days after FMT, which is considered unlikely to be related to FMT; both patients recovered spontaneously. No other serious adverse events were reported. Of the 9 baseline-AIEC-positive patients, 8 completed follow-up, and all showed mucosal AIEC clearance at week 12 (100%; Fig. 1b). All participants who were AIEC-negative at baseline remained AIEC-negative at week 12. Following FMT, overall disease activity remained stable with no significant changes in HBI or CDAI after FMT. Some laboratory parameters showed changes compared with baseline, including a decrease in WBC count (*P* = 0.0001), platelet count (*P* = 0.048) and alkaline phosphatase (*P* = 0.002). Other clinical and laboratory indicators did not show significant changes (Table 2). Subgroup analyses stratified by baseline AIEC colonization are summarized in Supplementary Table 1. In patients who were AIEC-positive at baseline, HBI did not change significantly after FMT (Δ = −0.62, *P* = 0.095). In contrast, patients who were AIEC-negative at baseline showed a significant increase in CDAI after FMT (Δ = 16.08, *P* = 0.034). Between-group comparisons demonstrated significant differences in both ΔHBI (*P* = 0.040) and ΔCDAI (*P* = 0.027), indicating distinct clinical responses according to AIEC status.

**Table 2:** Changes of disease activity and laboratory parameters before and 12 weeks after FMT in all paired patients (*n* = 25)

Characteristics	Baseline	Follow-up	Δ	*P* value
**Disease activity scores**				
HBI	0.8 (1.0)	0.7 (1.1)	−0.04 (0.89)	0.824
CDAI	48.6 (32.3)	58.7 (34.0)	10.05 (25.77)	0.063
**Hematology markers**				
Hb (g/dL)	14.0 (1.4)	13.9 (1.5)	−0.09 (0.64)	0.497
RBC (× 10^12^/L)	4.9 (0.7)	4.8 (0.7)	−0.03 (0.22)	0.437
WBC (× 10^9^/L)	6.6 (1.7)	5.8 (1.6)	−0.84 (0.94)	0.0001
PLT (× 10^9^/L)	298.5 (68.3)	282.1 (62.7)	−16.40 (39.4)	0.048
**Inflammatory markers**				
Fecal Calprotectin (µg/g)	643.5 (813.4)	3175.0 (8317.2)	2531.43 (7845.68)	0.120
CRP (mg/L)	2.9 (5.4)	2.7 (5.1)	**−**0.19 (1.83)	0.613
ESR (mm/h)	18.0 (14.3)	18.2 (12.7)	0.2 (4.44)	0.824
**Biochemical indicators**				
Albumin (g/L)	39.9 (4.2)	39.4 (3.2)	−0.44 (2.52)	0.391
ALP (U/L)	69.4 (16.9)	64.4 (15.2)	−5.0 (7.31)	0.002
ALT (U/L)	21.7 (10.5)	24.8 (11.3)	3.16 (9.55)	0.111
TB (µmol/L)	10.0 (5.9)	14.0 (6.5)	3.96 (3.48)	0.000007
Creatinine (µmol/L)	74.5 (11.4)	73.7 (12.9)	−0.80 (7.42)	0.595
Potassium (mmol/L)	3.9 (0.3)	3.8 (0.3)	−0.12 (0.40)	0.161
Sodium (mmol/L)	140.2 (1.6)	138.8 (2.1)	−1.32 (2.27)	0.008

Abbreviations: *FMT* fecal microbiota transplantation; *HBI* Harvey-Bradshaw Index; *CDAI* Crohn’s Disease Activity Index; *Hb* Hemoglobin; *RBC* Red Blood Cell count; *WBC* White Blood Cell count; *PLT* Platelets; *CRP* C-Reactive Protein; *ESR* Erythrocyte Sedimentation Rate; *ALP* Alkaline Phosphatase; *ALT* Alanine Aminotransferase; *TB* Total Bilirubin.

Δ = Post-FMT - Pre-FMT. The *P* values were calculated using a two-sided paired t-test.

#### Fecal microbiome changes after FMT

**Fig. 2:**
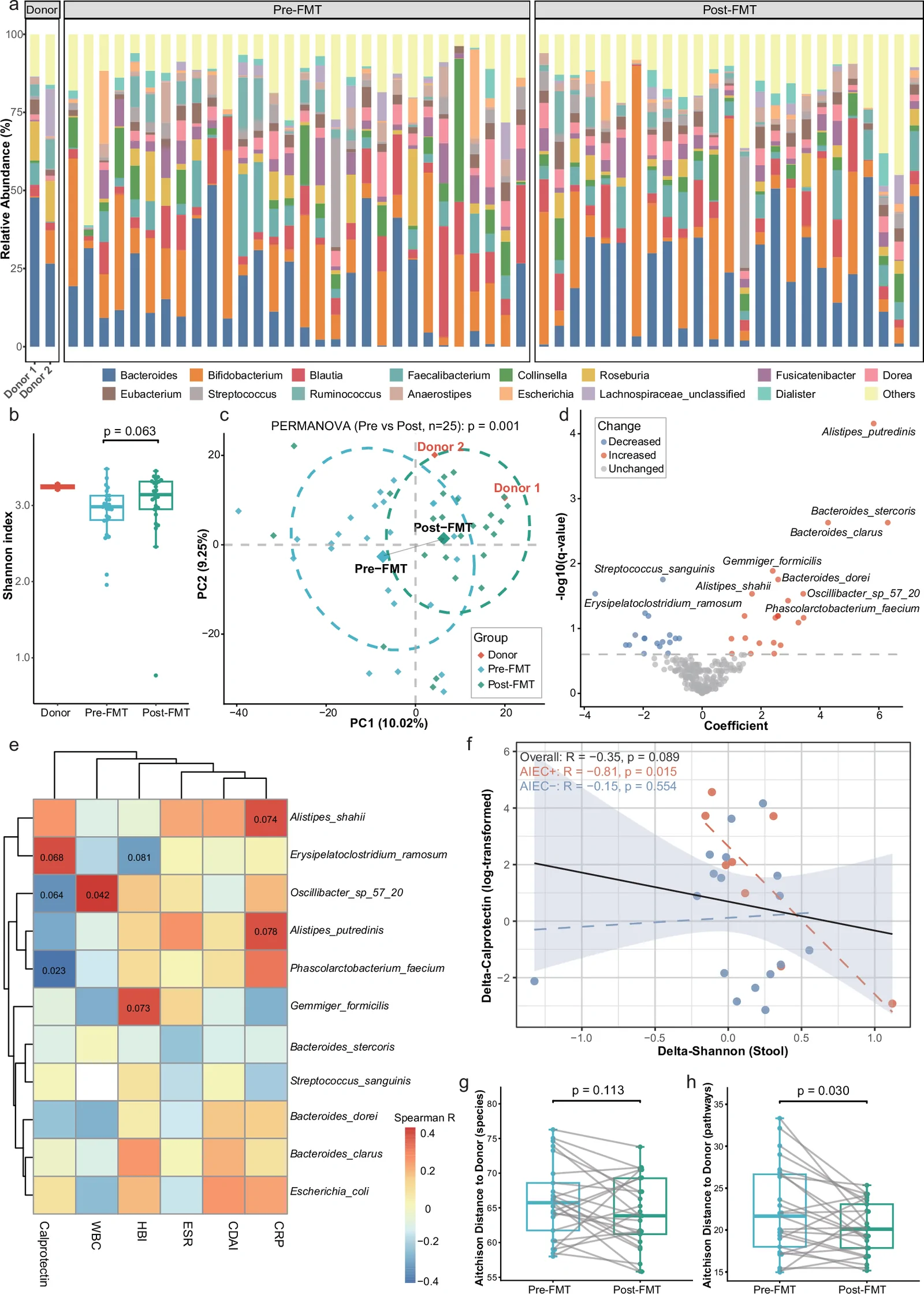
Global and donor-directed remodeling of the fecal microbiome after FMT and its associations with disease activity and inflammation. **a** Genus-level fecal microbiota composition in donors and recipients before and after FMT, showing the 15 most abundant genera; others are grouped as Others. **b** Fecal Shannon diversity before and after FMT (*n* = 25). **c** PCoA of paired fecal samples based on Aitchison distance (*n* = 25); group centroids and confidence ellipses are shown. Differences were assessed using PERMANOVA. **d** Differentially abundant fecal microbial species after FMT. MaAsLin2 was applied to paired samples (*n* = 25), with subject as a random effect. Red, blue and gray indicate increased, decreased and unchanged species, respectively. Species with FDR-adjusted *P* value (*q*) <0.05 are labeled. **e** Two-sided Spearman correlations between changes in CLR-transformed FMT-responsive species and clinical/inflammatory indices. Cell colors indicate correlation coefficients, and numbers indicate *P* values. **f** Association between changes in fecal Shannon diversity and fecal calprotectin by baseline mucosal AIEC status. The solid line and shaded area indicate the overall linear regression fit and its 95% confidence interval, respectively; dashed lines indicate subgroup linear regression fits. Two-sided Spearman *R* and *P* values are shown. **g**, **h** Donor-recipient Aitchison distances before and after FMT based on CLR-transformed species (**g**) and MetaCyc pathway (h) profiles from 25 recipients with paired pre- and post-FMT fecal metagenomes; gray lines connect paired samples. For b, g and h, *P* values were calculated using a two-sided paired Wilcoxon signed-rank test. Box plots show the median (center line), 25th-75th percentiles (box), and the most extreme values within 1.5 times the interquartile range (whiskers); points represent individual participants. FMT fecal microbiota transplantation; PCoA principal coordinate analysis; PERMANOVA permutational multivariate analysis of variance; MaAsLin2 Microbiome Multivariable Association with Linear Models; FDR false discovery rate; CLR centered log-ratio; AIEC adherent-invasive *E. coli*.

A total of 57 fecal samples were analyzed, including 2 donor samples and 55 recipient samples collected before (*n* = 30) and after FMT (*n* = 25). The most prominent difference between donors was a significantly higher abundance of *Bifidobacterium* in donor 2 (10.7%) versus donor 1 (0.4%) (Fig. 2a), primarily driven by *Bifidobacterium adolescentis* (Supplementary Fig. 1). In paired recipients (*n* = 25), fecal Shannon diversity did not differ significantly before and after FMT (*P* = 0.063, Fig. 2b), whereas the unpaired analysis of all samples reached significance (*P* = 0.038, Supplementary Fig. 2). Additional alpha diversity metrics, including Observed Richness and Pielou’s Evenness, were also evaluated and did not show significant changes following FMT (Supplementary Fig. 3). Fecal community structure significantly differed between pre- and post-FMT samples (PERMANOVA, *P* = 0.001, Fig. 2c and Supplementary Fig. 4).

Species-level differential abundance was assessed using MaAsLin2 in paired samples (*n* = 25) with ‘subject’ as a random effect. The abundance of several taxa was significantly altered after FMT, including decrease in *Erysipelatoclostridium ramosum* (MaAsLin coef = −3.6) and *Streptococcus sanguinis* (MaAsLin coef = −1.3) and increase in *Alistipes putredinis* (MaAsLin coef = 5.8), *Gemmiger formicilis* (MaAsLin coef = 2.4), *Phascolarctobacterium faecium* (MaAsLin coef = 2.9), and *Alistipes shahii* (MaAsLin coef = 1.7) (Fig. 2d). This was accompanied by several significant changes in MetaCyc pathways (Supplementary Fig. 5). We further computed Spearman correlations between changes in microbiome features and inflammatory indices and found that the decrease in fecal calprotectin was associated with an increase in *Phascolarctobacterium faecium* (*P* = 0.023) (Fig. 2e). In addition, increase in fecal Shannon diversity was significantly correlated with reduction in fecal calprotectin in recipients who cleared mucosal AIEC (*R* = −0.81, *P* = 0.015), but not in those who remained AIEC negative (*R* = −0.15, *P* = 0.554) (Fig. 2f).

We next assessed the convergence of recipients toward their respective donors after FMT by comparing patient-donor dissimilarity (Aitchison distance) at baseline and week 12. At the species level, there was no significant reduction in donor dissimilarity after FMT (Wilcoxon signed-rank test, *P* = 0.113; Fig. 2g). In contrast, at the functional level, patient-donor dissimilarity decreased significantly, indicating a shift toward the donor (Wilcoxon signed-rank test, *P* = 0.030; Fig. 2h). Notably, recipients who cleared mucosal AIEC exhibited a significant reduction in pathway-level patient-donor dissimilarity (*P* = 0.039; Supplementary Figs. 6 and 7).

#### Mucosal microbiome remodeling after FMT

**Fig. 3:**
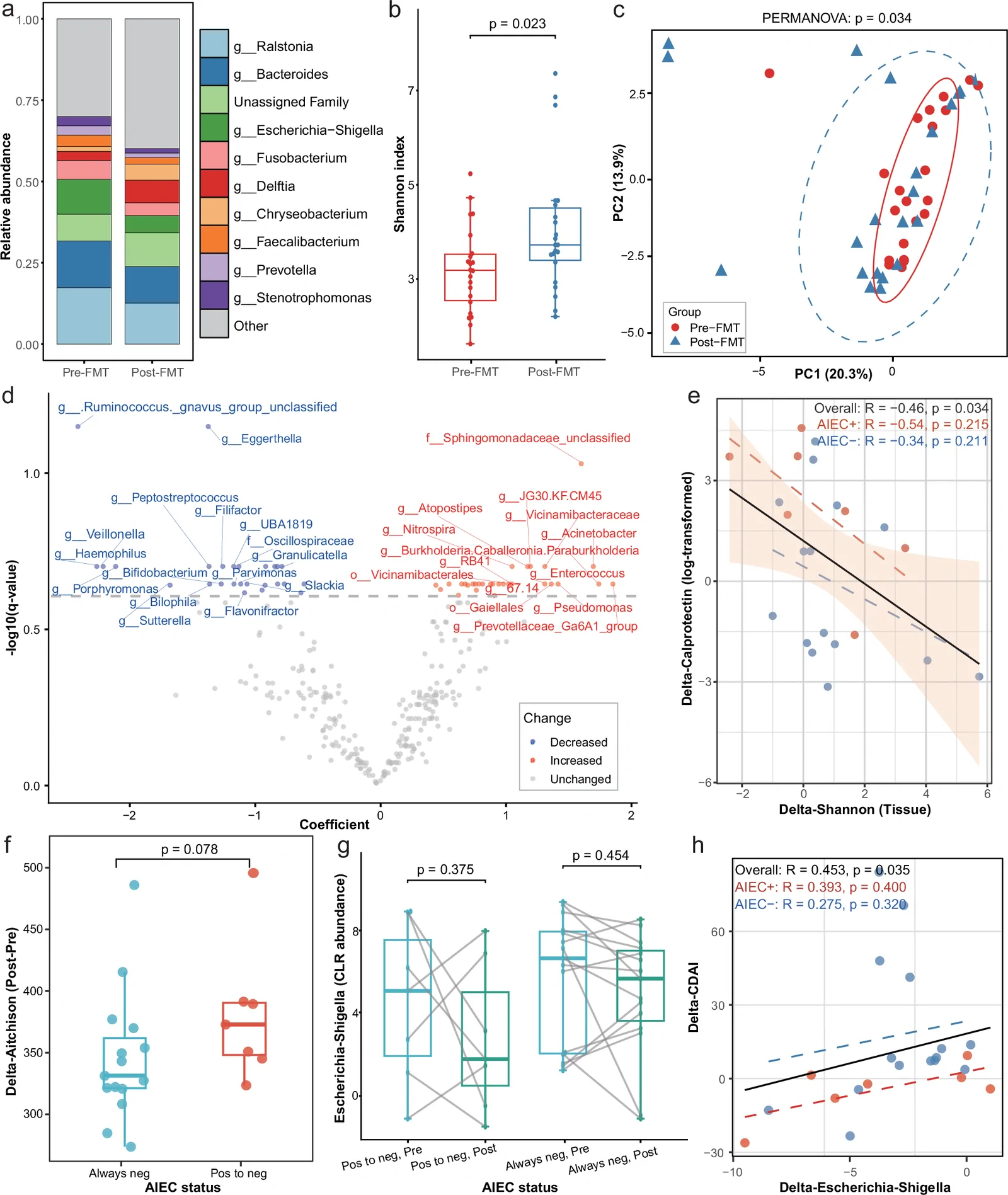
Mucosal microbiome shifts after FMT reveal AIEC-associated remodeling patterns and links to inflammation and disease activity. **a** Genus-level mucosal microbiota composition before and after FMT, showing the 10 most abundant genera; others are grouped as Other. **b** Mucosal Shannon diversity before and after FMT (*n* = 22). **c** PCoA of paired mucosal samples based on Aitchison distance (*n* = 22); confidence ellipses are shown. Differences were assessed using PERMANOVA. **d** Differentially abundant mucosal genera after FMT. MaAsLin2 was applied to paired samples (*n* = 22), with subject as a random effect. Red and blue indicate increased and decreased genus, respectively; gray indicates unchanged genera. Genus with *q* < 0.25 and |coefficient| > 1 are labeled. **e** Association between changes in mucosal Shannon diversity and fecal calprotectin by baseline AIEC status. The solid line and shaded area indicate the overall linear regression fit and 95% confidence interval, respectively; dashed lines indicate subgroup fits. Two-sided Spearman *R* and *P* values are shown. **f** Mucosal microbiome remodeling by AIEC status change. Aitchison distance changes (Post-Pre) from CLR-transformed OTU profiles (*n* = 22) were compared between Always-negative (*n* = 15) and Positive-to-*n*egative (*n* = 7) groups. **g** CLR-transformed mucosal *Escherichia-Shigella* abundance before and after FMT, stratified by AIEC status change (Always-negative, *n* = 15; Positive-to-negative, *n* = 7); gray lines connect paired samples. **h** Association between changes in mucosal *Escherichia-Shigella* abundance and CDAI. The solid line and shaded area indicate the overall linear regression fit and 95% confidence interval, respectively; dashed lines indicate subgroup fits. Two-sided Spearman *R* and *P* values are shown. For **b** and g, *P* values were calculated using a two-sided paired Wilcoxon signed-rank test; for **f**, a two-sided Wilcoxon rank-sum test was used. Box plots show the median (center line), 25th-75th percentiles (box), and the most extreme values within 1.5 times the interquartile range (whiskers); points represent individual participants. AIEC adherent-invasive *E. coli*; FMT fecal microbiota transplantation; PCoA principal coordinate analysis; PERMANOVA permutational multivariate analysis of variance; MaAsLin2 Microbiome Multivariable Association with Linear Models; CLR centered log-ratio; OTU operational taxonomic unit; CDAI Crohn’s Disease Activity Index.

A total of 51 mucosal biopsy samples from recipients were analyzed, including 27 samples collected before FMT and 24 collected after FMT; paired mucosal samples were available in 22 recipients. Genus-level composition based on 16S rRNA profiling revealed shifts in several taxa after FMT, notably a reduction in *Escherichia-Shigella* (Fig. 3a and Supplementary Fig. 8). Mucosal Shannon diversity increased significantly after FMT (*n* = 22, Wilcoxon signed-rank test, *P* = 0.023; Fig. 3b) and in all available mucosal samples (Wilcoxon rank sum test, *P* = 0.009, Supplementary Fig. 9). Additional alpha diversity analyses showed a significant increase in Pielou’s evenness (*P* = 0.046), whereas Observed Richness (*P* = 0.058) and Faith’s Phylogenetic Diversity (*P* = 0.262) did not differ significantly following FMT (Supplementary Fig. 10). Mucosal community structure differed significantly after FMT (PERMANOVA, paired analysis, *P* = 0.034, Fig. 3c; unpaired *P* = 0.083, Supplementary Fig. 11).

To identify differential genera associated with FMT, we performed MaAsLin2 in paired mucosal samples (*n* = 22) with the subject as a random effect. Several genera decreased after FMT, including *Ruminococcus gnavus group (unclassified)* (MaAsLin coef = −2.4), *Eggerthella* (MaAsLin coef = −1.3), *Haemophilus* (MaAsLin coef = −2.2), *Sutterella* (MaAsLin coef = −1.6), and *Bilophila* (MaAsLin coef = −1.3), whereas genera such as *Prevotellaceae Ga6A1 group* (MaAsLin coef = 1.9) and *Enterococcus* (MaAsLin coef = 1.3) increased (Fig. 3d). Notably, changes in *Escherichia-Shigella* were positively correlated with changes in CDAI (Supplementary Fig. 12). Additional significant correlations were observed for *Sutterella* with HBI and WBC, *Ruminococcus gnavus* group with ESR, *Bilophila* with ESR, *Haemophilus* with CDAI, and *Enterococcus* with CRP (Supplementary Fig. 12). Mucosal Shannon diversity changes were inversely associated with fecal calprotectin changes in the overall cohort (*P* = 0.034; Fig. 3e), with no significant associations within AIEC subgroups.

We then evaluated mucosal microbiome remodeling in relation to AIEC status change. No significant difference in Aitchison distance change was observed between recipients who cleared AIEC and those who remained AIEC negative (Wilcoxon rank-sum test, *P* = 0.078; Fig. 3f). Similarly, mucosal *Escherichia-Shigella* abundance did not change significantly after FMT (Fig. 3g). Nevertheless, a decrease in mucosal *Escherichia-Shigella* was correlated with a decrease in CDAI (Spearman correlation, *P* = 0.035; Fig. 3h and Supplementary Fig. 13).

### Species-level engraftment and multidimensional donor effects

**Fig. 4:**
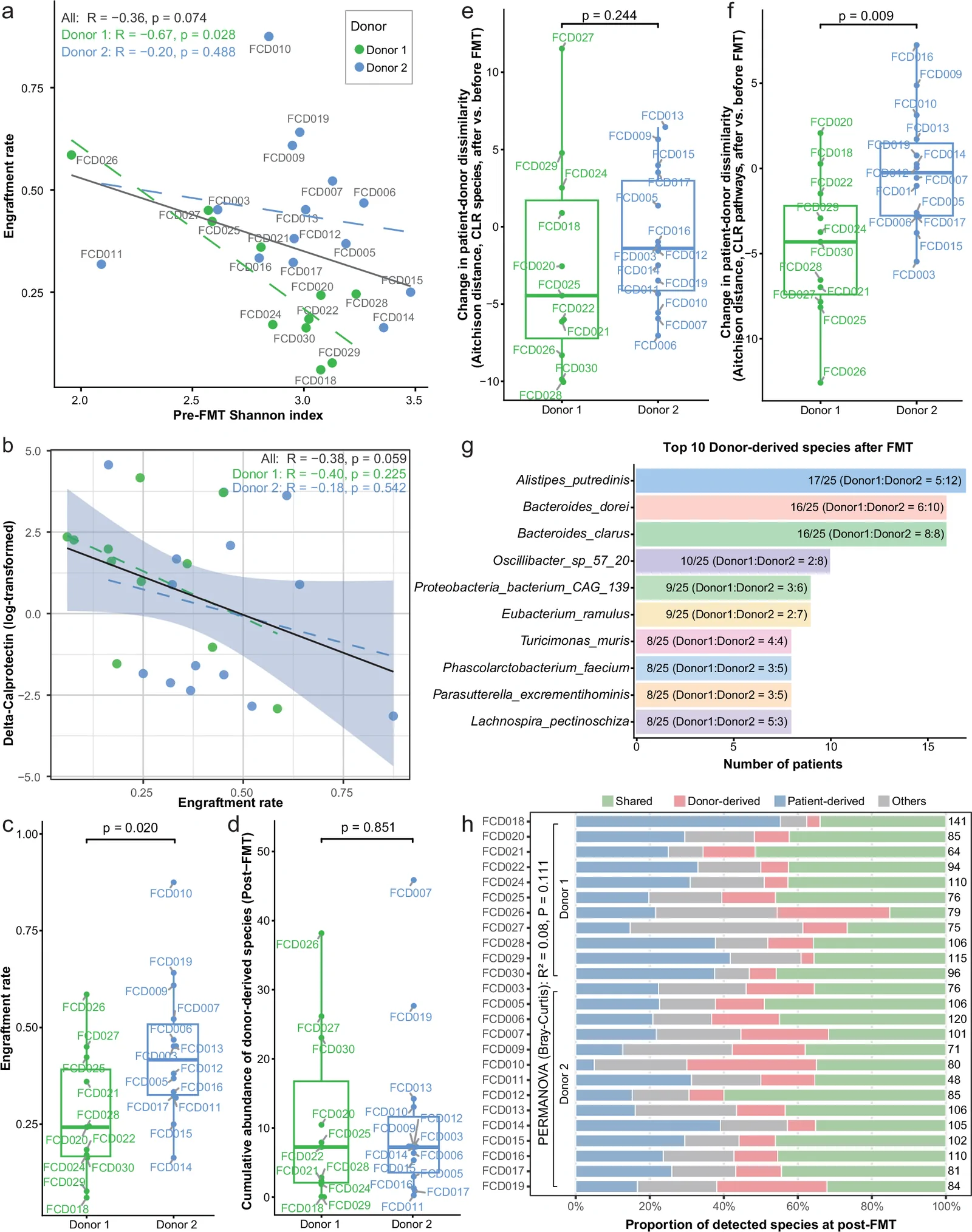
Species-level engraftment and multidimensional donor effects after FMT. **a** Association between species-level donor engraftment and pre-FMT fecal Shannon diversity. Two-sided Spearman *R* and *P* values are shown for the overall cohort and each donor group. **b** Association between engraftment rate and change in fecal calprotectin (log-transformed). The solid line and shaded area indicate the overall linear regression fit and 95% confidence interval, respectively; dashed lines indicate donor-specific fits. Two-sided Spearman *R* and *P* values are shown. **c** Species-level engraftment rate by donor, calculated as Spd/(Spp + Spd), where Spd and Spp denote donor-derived and patient-derived species, respectively. **d** Cumulative relative abundance of donor-derived species post-FMT by donor. **e**, **f** Changes in recipient-donor Aitchison distance after FMT based on CLR-transformed species (**e**) and MetaCyc pathway (**f**) profiles; negative values indicate increased donor similarity. For **c**–**f**, donor groups (Donor 1, *n* = 11; Donor 2, *n* = 14) were compared using two-sided Wilcoxon rank-sum tests. Box plots show the median (center line), 25th-75th percentiles (box), and the most extreme values within 1.5 times the interquartile range (whiskers); points represent individual recipients. **g** Top 10 donor-derived species at Week 12 (*n* = 25), ranked by recipient prevalence; labels show the total count and distribution by donor. **h** Post-FMT species source composition by donor (*n* = 25). Species were classified as donor-derived, patient-derived, shared (present pre-FMT, post-FMT and in the donor), or others. Bars show their proportions, with total species counts indicated on the right. Donor groups were compared using PERMANOVA based on Bray-Curtis distance. FMT fecal microbiota transplantation; CLR centered log-ratio; PERMANOVA permutational multivariate analysis of variance.

Species-level donor engraftment varied markedly across recipients. Lower baseline fecal Shannon index was not significantly associated with species-level engraftment rate in the overall cohort (Spearman’s test, *P* = 0.074; Fig. 4a). When stratified by donor, this inverse relationship was significant among recipients of donor 1 (*P* = 0.028) but not among recipients of donor 2 (Fig. 4a), indicating that baseline ecological resistance to engraftment was more pronounced in the donor 1 recipient group.

Baseline inflammatory status, as assessed by fecal calprotectin levels, was not significantly associated with subsequent species-level engraftment rate, either overall or when stratified by donor (Supplementary Fig. 14), suggesting that baseline inflammation did not substantially constrain donor microbial engraftment. In addition, the association between species-level engraftment rate and changes in fecal calprotectin did not reach statistical significance (log-transformed Δcalprotectin, Spearman’s test, *P* = 0.059; Fig. 4b).

Notably, a clear donor-specific effect was observed. Recipients of donor 2 exhibited a significantly higher species-level engraftment rate than recipients of donor 1 (Wilcoxon rank-sum test, *P* = 0.020; Fig. 4c). However, this difference in engraftment richness did not translate into greater cumulative relative abundance of donor-derived species in the recipients’ post-FMT samples (Fig. 4d). Similarly, donor-directed convergence at the species level did not differ significantly between the two groups (*P*= 0.244; Fig. 4e). This indicates that a higher number of donor-derived species did not equate to greater occupation within the recipient’s reconstituted microbiota. In contrast, recipients of donor 1 showed a greater reduction in dissimilarity based on MetaCyc pathway profiles compared with recipients of donor 2 (*P* = 0.009; Fig. 4f), indicating stronger functional convergence toward the donor despite lower species-level engraftment.

Finally, taxon-level patterns of origin assignment were summarized by the most frequently detected donor-derived species at week 12. The top donor-derived species included *Alistipes putredinis*, *Eubacterium ramulus*, *Lachnospira pectinoschiza*, and *Phascolarctobacterium faecium* (Fig. 4g), all of which are typically reported to be reduced in patients with CD and produce short-chain fatty acids with potential anti-inflammatory effects, contributing to gut barrier maintenance and modulation of intestinal inflammation. Post-FMT species composition at week 12 varied across recipients but was not significantly influenced by donor assignment (PERMANOVA, *P* = 0.111, Fig. 4h). Stratification by donor revealed no differences in donor-, patient-, or shared-species proportions, except for a donor-dependent difference in the ‘others’ category (*P* = 0.031, Supplementary Fig. 15).

## Discussion

In this open-label pilot study, FMT was associated with mucosal AIEC decolonization in CD patients. To our knowledge, this is the first human study to report the impact of FMT on AIEC colonization. In addition, mucosal alpha diversity increased after FMT, an effect not observed in fecal samples. These findings demonstrate the potential of FMT to modulate mucosal AIEC colonization and highlight the importance of capturing microbial changes at the disease site that may be undetectable in fecal samples.

Notably, AIEC was undetectable in all evaluable baseline-positive AIEC patients. This clearance was associated with the convergence of microbial functional profiles toward that of the donor rather than with simple taxonomic replacement. This observation suggests that donor-derived ecological and metabolic functions, such as bile acid transformation and short-chain fatty acids production, rather than individual antagonistic taxa, may create a mucosal environment unfavorable for AIEC persistence^23–25^. While our previous animal models indicated that AIEC could resist FMT clearance^12^, the effective clearance observed in our patients may be attributed to a more permissive host environment in the clinical setting. The murine model employed an induced colitis model with sustained AIEC colonization, creating an inflamed, dysbiotic “soil” that may inherently resist the engraftment and functional expression of donor microbiota. In contrast, our patient cohort was predominantly mild or in clinical remission, presenting mucosa with reduced inflammation and a more anaerobic, ecologically complex environment. This “healthier soil” is likely more conducive to the durable engraftment and functional activity of donor-derived microbes. Importantly, exploratory subgroup analyses suggested distinct patterns of disease activity measures according to baseline AIEC status, suggesting that the microbiological effects of FMT may be influenced by the baseline intestinal environment of the recipient, with a less inflamed mucosa potentially providing conditions more favorable for donor microbiota engraftment and colonization resistance.

Beyond AIEC clearance, we also observed compositional shifts in both fecal and mucosal microbiomes. In fecal samples, pro-inflammatory taxa such as *Erysipelatoclostridium ramosum*^26^ and the *Ruminococcus gnavus* group^27^ decreased, while butyrate-producing species such as *Phascolarctobacterium faecium*^28^ and *Gemmiger formicilis*^29^ increased. In mucosal samples, reductions were observed in mucosa-associated pathobionts such as *Haemophilus*^30^ and *Bilophila*^31^. Importantly, a significant increase in alpha diversity following FMT was observed only in ileal mucosal samples but not in fecal samples. The increased ileal mucosal microbial diversity was associated with reduced fecal calprotectin, and changes in mucosal *Escherichia-Shigella* abundance correlated with changes in disease activity scores. Reduced microbial diversity is a well-recognized hallmark of CD^32^, supporting a link between restored mucosal diversity and attenuation of intestinal inflammation. As mucosal communities reside at the host interface and more directly reflect host-microbe interactions^33^, the observed mucosal-specific increases in diversity and reductions in pathobionts suggest that FMT is associated with broader ecological changes at the disease site. This pattern aligns with recent findings in oncology, where FMT success was primarily driven by the elimination of baseline inhibitory bacteria rather than the simple engraftment of donor strains^34^. By extension, our results suggest that the efficacy of FMT in CD may depend more on resetting the mucosal environment by purging pathobionts than on the mere addition of donor taxa. Monitoring these ecological changes within the mucosal compartment may therefore offer a more direct and biologically relevant way to evaluate FMT response than fecal sampling alone.

Patients with baseline mucosal AIEC colonization exhibited significantly higher serum AEcAb levels, which did not decline following successful AIEC clearance. This indicates that AEcAb reflects long-standing immune memory rather than current mucosal bacterial burden. Consistent with observations that antibody responses can persist independently of ongoing antigen presence^35,36^. Thus, AEcAb may represent a stable immunological phenotype marker rather than a short-term biomarker of treatment response.

In most recipients, post-FMT microbial communities generally became more similar to their respective donors, with all individuals exhibiting a measurable donor-derived species engraftment. Engraftment was influenced by both donor and recipient factors: recipients of donor 2 exhibited higher engraftment rates than those of donor 1, while higher baseline alpha diversity was negatively associated with engraftment rate among recipients of donor 1. This is consistent with previous reports that a more diverse resident microbiota constrains donor colonization^37–39^. Notably, we observed a clear dissociation: higher taxonomic engraftment did not translate into greater functional convergence. These findings indicate that successful engraftment does not necessarily translate into functional integration, supporting the concept of functional redundancy, whereby substantial functional reconfiguration can occur in the absence of marked changes in species composition^40^.

Several important limitations merit careful consideration. First, this study was designed as an exploratory proof-of-concept pilot study with no control group and a small sample size, which precludes direct attribution of observed changes to FMT or causal inferences. Second, the cohort mainly included patients in clinical remission or with mild disease activity. While this was purposely designed to provide a favorable “soil” for donor microbiota engraftment—as an actively inflamed gut may inherently resist functional engraftment—it limited the assessment of overt clinical improvement. Third, endoscopic endpoints were not included and should be incorporated in future studies to directly link AIEC clearance with mucosal healing. Fourth, AIEC decolonization refers to the absence of detectable AIEC rather than proof of absolute eradication, as the detection method is inherently limited by sampling and technical sensitivity. Nevertheless, the direct assessment of mucosal AIEC colonization is a key strength of this study, as sequencing-based methods cannot distinguish AIEC from commensal *E. coli*. Finally, the 12-week follow-up duration limits the assessment of the long-term durability of post-FMT decolonization. Future studies with proper controls, larger sample sizes, and extended observation periods are warranted to better characterize AIEC dynamics and evaluate long-term outcomes.

In conclusion, this open-label pilot study demonstrates the feasibility of FMT for modulating mucosal AIEC colonization and remodeling the ileal mucosal microbiome in patients with CD, and highlights the importance of capturing microbial changes at the disease site that may be undetectable in fecal samples.

## Methods

### Study design

This was a single-center, open-label pilot study conducted between October 2022 and January 2023 to evaluate the safety of FMT in adults with CD at risk of AIEC colonization, as indicated by seropositivity for AEcAb. After eligibility screening, baseline assessments included disease activity indices (Harvey-Bradshaw Index, HBI; Crohn’s Disease Activity Index, CDAI), routine laboratory tests, and collection of stool and ileal mucosal biopsies for microbiome profiling and AIEC assessment. All participants received a single FMT infusion via colonoscopy (Day 0). The primary evaluation timepoint was at follow-up after FMT (Week 12). Stool samples for fecal calprotectin were collected at screening and at follow-up visit; repeated ileal biopsies were obtained at Week 12 when feasible.

The primary outcome was the occurrence of adverse events (AEs) during and within 12 weeks after FMT, graded using the Common Terminology Criteria for Adverse Events (CTCAE) v4.03. Transient symptoms resolving within 3 days were not considered AEs unless persistent or worsening. All AEs were reviewed by an independent adjudication committee comprising clinicians and microbiologists. Secondary outcomes included changes in mucosal AIEC, clinical and laboratory measures, and fecal and mucosal microbiome profiles (taxonomic and functional).

The study protocol was approved by the Ethics Committee of the Chinese University of Hong Kong (CREC Ref. No.: 2014.026) and was conducted in accordance with the Declaration of Helsinki and International Council for Harmonization Good Clinical Practice (ICH-GCP) guidelines. All participants provided written informed consent prior to any study procedures. Participants did not receive compensation for participation in the study. The trial was registered at ClinicalTrials.gov (NCT05611866). The study protocol is provided with this article.

### Study population

Patients who met the following criteria were included in the study: (1) age >18 years; (2) confirmed diagnosis of CD with a disease duration of at least 3 months; (3) no or mild-to-moderate symptoms, defined as a HBI score <16; and (4) seropositivity for AEcAb. The exclusion criteria were as follows: (1) pregnancy; (2) initiation of a new biologic therapy or corticosteroid use within the previous 4 weeks; (3) presence of colorectal tumor; (4) active gastrointestinal bleeding; (5) diagnosis of ulcerative colitis; (6) history of colectomy or partial colectomy with less than ileo-transverse colonic anastomosis; (7) presence of a colonic or small bowel stoma; (8) active perianal lesions; (9) use of antibiotics within the previous 4 weeks; and (10) psychological or linguistic incapacity to provide informed consent.

### Stool donors and FMT protocol

Stool used for FMT was sourced from the Chinese University of Hong Kong stool bank established under ethics-approved donor-screening protocols (CREC Ref. No.: 2016.033 and 2016.707). Healthy adult volunteers from the community (including relatives and unrelated individuals) were screened using standardized questionnaires and laboratory testing according to the stool bank protocol. Eligible donors were required to have a body mass index (BMI) ≥ 18.5 and <23 kg/m^2^, normal hematology and biochemistry results, no known gastrointestinal, metabolic, chronic, malignant, or infectious diseases, and no evidence of enteric or blood-borne pathogens on screening tests. Donors also had no recent exposures or medications that would preclude donation as defined by the stool bank protocol. Stool from two eligible donors (donor 1 and donor 2) was used in this study. For each FMT infusion, the preparation was derived from a single donor (donor 1 or donor 2). Briefly, 50–100 g of stool was resuspended in 250–350 mL of sterile 0.9% sodium chloride, homogenized using a single-use blender, and filtered through sterile non-woven gauze to obtain a filtered fecal suspension. The filtered preparation designated for infusion was adjusted to a final volume of 150 mL with sterile saline and administered via colonoscopy into the terminal ileum.

### Measurement of AEcAb

AEcAb levels were quantified using an in-house enzyme-linked immunosorbent assay (ELIZA)^35^. Briefly, 96-well plates were coated with *E.coli MG1655* (10^7^ CFU/well) by overnight drying at 37 °C. After washing with phosphate-buffered saline (PBS) containing 0.05% Tween-20 (PBST), the plates were blocked with 5% skim milk in PBS for 2 h at room temperature. Patient serum samples (50 μL) and positive controls (Human AB serum, Sigma-Aldrich, H4522) were serially diluted (1/4 to 1/95367.43) in blocking buffer and incubated for 2 h at room temperature. Following three washes with PBST, the plates were incubated for 2 h with horseradish peroxidase (HRP)-conjugated goat anti-human IgG secondary antibody (Sigma-Aldrich; diluted 1:50,000) at room temperature. After another washing step, the reaction was developed by adding 3,3’,5,5’-tetramethylbenzidine (TMB) substrate solution and incubating for 40 min. The reaction was stopped by adding 50 μL stop solution (1 M H_2_SO_4_), and the absorbance was immediately measured at 450 nm with a reference wavelength of 570 nm. The half-maximal effective concentration (EC_50_) titer for each sample was calculated by four-parameter nonlinear regression analysis using GraphPad Prism software, with values normalized to the included controls.

### Identification of AIEC

Mucosal AIEC colonization was identified using established methods^41^. Ileal biopsy tissues obtained during colonoscopy were immediately immersed in Minimum Essential Medium (MEM) supplemented with 15% sterile glycerol, snap-frozen in liquid nitrogen, and stored at −80 °C until further processing. *E.coli* adherent to the intestinal mucosa and invading intestinal epithelial cells were isolated from the preserved biopsy suspensions and lysates, and colony-forming units per milligram (CFU/mg) were determined on Drigalski agar. From each sample, a maximum of 45 *E.coli* colonies were randomly selected for further analysis (fewer were analyzed if 45 colonies were not isolated). The invasive ability of each isolate was confirmed using a gentamicin protection assay with Intestine-407 cells (ATCC® CCL-6™). The prototype AIEC strain LF82 and a non-invasive control strain K12C600 were included as references in each assay. The patients were considered AIEC positive if the isolated *E. coli* strains had an invasion rate equal to or superior to 10% of LF82.

### Clinical/laboratory parameters

Clinical and laboratory assessments were conducted at two time points: baseline and the follow-up visit between Week 12 (within a 7-day window). Vital signs were recorded at both timepoints. Disease activity was assessed using HBI and CDAI. Laboratory evaluations included complete blood count, including white blood cell (WBC), red blood cell (RBC), hemoglobin, and platelets; renal function markers (creatinine, potassium, sodium); liver function tests (albumin, alanine aminotransferase, alkaline phosphatase, total bilirubin); as well as C-reactive protein (CRP) and erythrocyte sedimentation rate (ESR). Serum AEcAb levels were also measured.

### Fecal metagenomic sequencing

Stool samples were collected at baseline and week 12. Fecal DNA for bacterial shotgun metagenomics sequencing was extracted using Maxwell® RSC PureFood GMO and Authentication Kit according to the manufacturer’s protocol. DNA libraries were constructed through the processes of end repairing, purification, and PCR amplification, and sequenced by Illumina Novaseq 6000 with a paired-end 150 bp sequencing strategy by Novogene, Beijing, China, generating 49.2 ± 7.3 million (mean ± standard deviation) raw reads per sample. Raw reads were quality-filtered and trimmed using Trimmomatic19 (v0.39) and decontaminated against the human genome (Reference: hg38) by Kneaddata (v0.7.2). Raw sequencing data are available at NCBI under Bioproject PRJNA1401743 and PRJNA1401742.

#### Donor species engraftment

Species-level engraftment was quantified using paired fecal samples from recipients with available baseline (pre-FMT) and week-12 (post-FMT) metagenomes and the corresponding donor metagenome. For each recipient, species detected in the post-FMT sample were compared with those in the recipient’s baseline sample and the assigned donor sample. Donor-derived species (Spd) were defined as species present in both the post-FMT sample and the donor sample but absent from the recipient’s baseline sample. Patient-derived species (Spp) were defined as species present in both the post-FMT and baseline samples but absent from the donor sample. The species-level engraftment rate was calculated as Spd/(Spd+Spp), where Spd and Spp represent the numbers of donor-derived and patient-derived species, respectively. Species shared between the donor and the recipient at baseline and species with unknown origin (present post-FMT but absent from both donor and baseline) were not included in the engraftment calculation. Engraftment analyses were performed on MetaPhlAn-derived species tables.

### Mucosa microbiota analysis

Microbial DNA was extracted from ileal mucosa biopsy samples. Each biopsy was combined with lysis buffer and zirconia beads and homogenized using a tissuelyser. After centrifuging the biopsy lysate, the supernatant was collected and then added to the Maxwell Tissue DNA Purification kit (Promega, Wisconsin, USA). The Maxwell RSC instrument was used for the purification of mucosal DNA. The extracted DNA was sequenced in the Novogene company, targeting the V3-V4 region of the 16S rRNA gene using primers 341 F (CCTAYGGGRBGCASCAG) and 806 R (GGACTACNNGGGTATCTAAT). The Illumina Novaseq 6000 platform (Illumina, San Diego, CA) was used for sequencing to generate PE 250 bp raw reads. The DNA extraction and sequencing method were described in the previous study^12^. Sequence data were pre-processed using QIIME2 (v2024.10) to remove primers and low-quality bases. Raw reads were clustered by VSEARCH, and those reads with 97% similarity were used to generate the Operational Taxonomic Units (OTUs). The taxonomy of OTUs was assigned by the SILVA 138 classifier that was trained using the Naïve Bayes algorithm^42^. We collapsed all the OTUs to the genus level to minimize the batch effect in sequencing^43^. Low-frequency genera with a total abundance of less than 0.01 of the abundance of all taxa were filtered from the OTUs table.

### Statistical analysis

All statistical analyses were conducted using R (v4.3.1) and GraphPad Prism (v8.0). Continuous variables are summarized as mean (standard deviation) and categorical variables as n (%). Between-group comparisons were performed using two-sided Welch’s t-test or Mann-Whitney U test, as appropriate; within-subject changes from baseline to week 12 were assessed using a paired t-test. Categorical outcomes were compared using chi-square or Fisher’s exact test. Microbiome analyses were conducted separately for fecal shotgun metagenomics and ileal mucosal 16S rRNA data. Species-level taxonomic profiling was performed using MetaPhlAn3 (v3.0.9), and functional profiling was conducted using HUMAnN3 (v3.0.0). Resulting abundance tables were processed using the tidyverse (v2.0.0) and phyloseq (v1.24.2) packages. Alpha diversity was assessed using the Shannon index. Beta diversity was evaluated using Aitchison distances derived from centered log-ratio (CLR)-transformed profiles and visualized by principal coordinate analysis (PCoA). Differential abundance analyses of taxa and pathways were performed using general linear model-based Microbiome Multivariable Association with Linear Models (MaAsLin2, v1.24.1). Benjamini-Hochberg false discovery rate (FDR) correction was applied, and features with FDR-adjusted *q* values < 0.25 were considered statistically significant. Associations between microbiome features and clinical or laboratory measures were evaluated using Spearman’s rank correlation. The engraftment rate was calculated as Spd/(Spd+Spp), excluding species shared at baseline and species with unknown origin. For analyses other than MaAsLin2 differential abundance testing, all tests were two-sided, and *P* < 0.05 was considered statistically significant.

### Reporting summary

Further information on research design is available in the Nature Portfolio Reporting Summary linked to this article.

## Supplementary information

Supplementary Information

Reporting Summary

Transparent Peer Review file

## Source data

Source Data

## Data Availability

The raw sequencing data generated in this study have been deposited in the NCBI Sequence Read Archive (SRA) under BioProject accession codes PRJNA1401743 and PRJNA1401742. The full results of the analyses, including all exact *P* values and de-identified subject-level clinical metadata, are provided in the Source Data file. The clinical metadata can be linked to the corresponding sequencing files. Source data are provided with this paper.

## References

[1] Torres, J., Mehandru, S., Colombel, J. F. & Peyrin-Biroulet, L. Crohn’s disease. *Lancet* **389**, 1741–1755 (2017). 10.1016/S0140-6736(16)31711-1 27914655

[2] Chhibba, T. et al. Environmental risk factors of inflammatory bowel disease: toward a strategy of preventative health. *J. Crohns Colitis* **19**, jjaf042 (2025). 10.1093/ecco-jcc/jjaf042 PMC12010164 40065502

[3] Liu, J. Z. et al. Association analyses identify 38 susceptibility loci for inflammatory bowel disease and highlight shared genetic risk across populations. *Nat. Genet.* **47**, 979–986 (2015). 10.1038/ng.3359 PMC4881818 26192919

[4] Lloyd-Price, J. et al. Multi-omics of the gut microbial ecosystem in inflammatory bowel diseases. *Nature* **569**, 655–662 (2019). 10.1038/s41586-019-1237-9 PMC6650278 31142855

[5] Ng, S. C. et al. Worldwide incidence and prevalence of inflammatory bowel disease in the 21st century: a systematic review of population-based studies. *Lancet* **390**, 2769–2778 (2017). 10.1016/S0140-6736(17)32448-0 29050646

[6] Pittayanon, R. et al. Differences in gut microbiota in patients with vs without inflammatory bowel diseases: a systematic review. *Gastroenterology* **158**, 930–946.e931 (2020). 10.1053/j.gastro.2019.11.294 31812509

[7] Palmela, C. et al. Adherent-invasive Escherichia coli in inflammatory bowel disease. *Gut.* **67**, 574–587 (2018). 10.1136/gutjnl-2017-314903 29141957

[8] Darfeuille-Michaud, A. et al. High prevalence of adherent-invasive Escherichia coli associated with ileal mucosa in Crohn’s disease. *Gastroenterology* **127**, 412–421 (2004). 10.1053/j.gastro.2004.04.061 15300573

[9] Bruder, E. et al. Persistence mechanisms of Crohn’s disease-associated adherent invasive Escherichia coli within macrophages. *Gut Microbes* **17**, 2587402 (2025). 10.1080/19490976.2025.2587402 PMC12931692 41287568

[10] Viladomiu, M. et al. IgA-coated E. coli enriched in Crohn’s disease spondyloarthritis promotes T(H)17-dependent inflammation. *Sci. Transl. Med.* **9**, eaaf9655 (2017). 10.1126/scitranslmed.aaf9655 PMC6159892 28179509

[11] Chassaing, B., Koren, O., Carvalho, F. A., Ley, R. E. & Gewirtz, A. T. AIEC pathobiont instigates chronic colitis in susceptible hosts by altering microbiota composition. *Gut.* **63**, 1069–1080 (2014). 10.1136/gutjnl-2013-304909 PMC4089387 23896971

[12] Zhilu, X. et al. Association of adherent-invasive Escherichia coli with severe gut mucosal dysbiosis in the Hong Kong Chinese population with Crohn’s disease. *Gut Microbes* **13**, 1994833 (2021). 10.1080/19490976.2021.1994833 PMC8632309 34812117

[13] Carvalho, F. A. et al. Crohn’s disease adherent-invasive Escherichia coli colonize and induce strong gut inflammation in transgenic mice expressing human CEACAM. *J. Exp. Med.* **206**, 2179–2189 (2009). 10.1084/jem.20090741 PMC2757893 19737864

[14] Schmitz, J. M. et al. Murine adherent and Invasive E. coli induces chronic inflammation and immune responses in the small and large intestines of mono-associated IL-10-/- Mice independent of long polar fimbriae adhesin A. *Inflamm. Bowel Dis.* **25**, 875–885 (2019). 10.1093/ibd/izy386 PMC6458545 30576451

[15] Baunwall, S. M. D. et al. Faecal microbiota transplantation for recurrent Clostridioides difficile infection: An updated systematic review and meta-analysis. *EClinicalMedicine* **29-30**, 100642 (2020). 10.1016/j.eclinm.2020.100642 PMC7788438 33437951

[16] Lopetuso, L. R. et al. The first international Rome consensus conference on gut microbiota and faecal microbiota transplantation in inflammatory bowel disease. *Gut.* **72**, 1642–1650 (2023). 10.1136/gutjnl-2023-329948 PMC10423477 37339849

[17] Peery, A. F. et al. AGA clinical practice guideline on fecal microbiota-based therapies for select gastrointestinal diseases. *Gastroenterology* **166**, 409–434 (2024). 10.1053/j.gastro.2024.01.008 38395525

[18] Costello, S. P. et al. Effect of fecal microbiota transplantation on 8-week remission in patients with ulcerative colitis: a randomized clinical trial. *JAMA* **321**, 156–164 (2019). 10.1001/jama.2018.20046 PMC6439766 30644982

[19] Zhou, S., Cui, Y., Zhang, Y., Zhao, T. & Cong, J. Fecal microbiota transplantation for induction of remission in Crohn’s disease: a systematic review and meta-analysis. *Int J. Colorectal Dis.* **38**, 62 (2023). 10.1007/s00384-023-04354-4 36882658

[20] Tian, H., Zhang, S., Qin, H., Li, N. & Chen, Q. Long-term safety of fecal microbiota transplantation for gastrointestinal diseases in China. *Lancet Gastroenterol. Hepatol.* **7**, 702–703 (2022). 10.1016/S2468-1253(22)00170-4 35709800

[21] Kao, D. et al. Preliminary results from a multicenter, randomized trial using fecal microbiota transplantation to induce remission in patients with mild-to-moderate Crohn’s disease. *Am. J. Gastroenterol.* **120**, 1334–1344 (2024). 10.14309/ajg.0000000000003196 PMC12124204 39530534

[22] Liu, C. S. et al. Towards optimising and standardising donor screening for fecal microbiota transplantation. *Gut*10.1136/gutjnl-2025-336532 (2025). 41298101

[23] Buffie, C. G. et al. Precision microbiome reconstitution restores bile acid-mediated resistance to Clostridium difficile. *Nature* **517**, 205–208 (2015). 10.1038/nature13828 PMC4354891 25337874

[24] Hamed, S. A. et al. Butyrate reduces adherent-invasive E. coli-evoked disruption of epithelial mitochondrial morphology and barrier function: involvement of free fatty acid receptor 3. *Gut Microbes* **15**, 2281011 (2023). 10.1080/19490976.2023.2281011 PMC10730202 38078655

[25] Winter, S. E. et al. Gut inflammation provides a respiratory electron acceptor for Salmonella. *Nature* **467**, 426–429 (2010). 10.1038/nature09415 PMC2946174 20864996

[26] Sheikh, I. A. et al. Transplant of microbiota from Crohn’s disease patients to germ-free mice results in colitis. *Gut Microbes* **16**, 2333483 (2024). 10.1080/19490976.2024.2333483 PMC10978031 38532703

[27] Hall, A. B. et al. A novel Ruminococcus gnavus clade enriched in inflammatory bowel disease patients. *Genome Med.* **9**, 103 (2017). 10.1186/s13073-017-0490-5 PMC5704459 29183332

[28] Dang, Y. et al. Gut microbiome signatures predict 5-ASA efficacy in ulcerative colitis. *iScience* **28**, 112568 (2025). 10.1016/j.isci.2025.112568 PMC12159923 40510115

[29] Ning, L. et al. Microbiome and metabolome features in inflammatory bowel disease via multi-omics integration analyses across cohorts. *Nat. Commun.* **14**, 7135 (2023). 10.1038/s41467-023-42788-0 PMC10628233 37932270

[30] Sohn, J. et al. Periodontal disease is associated with increased gut colonization of pathogenic Haemophilus parainfluenzae in patients with Crohn’s disease. *Cell Rep.* **42**, 112120 (2023). 10.1016/j.celrep.2023.112120 PMC10415533 36774550

[31] Devkota, S. et al. Dietary-fat-induced taurocholic acid promotes pathobiont expansion and colitis in Il10−/− mice. *Nature* **487**, 104–108 (2012). 10.1038/nature11225 PMC3393783 22722865

[32] Halfvarson, J. et al. Dynamics of the human gut microbiome in inflammatory bowel disease. *Nat. Microbiol* **2**, 17004 (2017). 10.1038/nmicrobiol.2017.4 PMC5319707 28191884

[33] Hu, S. et al. Mucosal host-microbe interactions associate with clinical phenotypes in inflammatory bowel disease. *Nat. Commun.* **15**, 1470 (2024). 10.1038/s41467-024-45855-2 PMC10874382 38368394

[34] Duttagupta, S. et al. Fecal microbiota transplantation plus immunotherapy in non-small cell lung cancer and melanoma: the phase 2 FMT-LUMINate trial. *Nat Med.* **32**, 1337–1350 (2026). 10.1038/s41591-025-04186-5 PMC13099432 41606121

[35] Buisson, A. et al. Faster and less invasive tools to identify patients with ileal colonization by adherent-invasive E. coli in Crohn’s disease. *U. Eur. Gastroenterol. J.* **9**, 1007–1018 (2021). 10.1002/ueg2.12161 PMC8598958 34791806

[36] Zinkernagel, R. M. & Hengartner, H. Protective ‘immunity’ by pre-existent neutralizing antibody titers and preactivated T cells but not by so-called ‘immunological memory. *Immunol. Rev.* **211**, 310–319 (2006). 10.1111/j.0105-2896.2006.00402.x 16824138

[37] Ianiro, G. et al. Variability of strain engraftment and predictability of microbiome composition after fecal microbiota transplantation across different diseases. *Nat. Med.* **28**, 1913–1923 (2022). 10.1038/s41591-022-01964-3 PMC9499858 36109637

[38] Podlesny, D. et al. Identification of clinical and ecological determinants of strain engraftment after fecal microbiota transplantation using metagenomics. *Cell Rep. Med.* **3**, 100711 (2022). 10.1016/j.xcrm.2022.100711 PMC9418803 35931074

[39] Zhao, D. et al. Recipients’ native bacteria determine the outcome of FMT treatment in inflammatory bowel disease. *Gut Microbes* **17**, 2600055 (2025). 10.1080/19490976.2025.2600055 PMC12710938 41399025

[40] Moya, A. & Ferrer, M. Functional redundancy-induced stability of gut microbiota subjected to disturbance. *Trends Microbiol.* **24**, 402–413 (2016). 10.1016/j.tim.2016.02.002 26996765

[41] O’Brien, C. L. et al. Comparative genomics of Crohn’s disease-associated adherent-invasive Escherichia coli. *Gut.* **66**, 1382–1389 (2017). 10.1136/gutjnl-2015-311059 27196580

[42] Robeson, M. S. et al. RESCRIPt: reproducible sequence taxonomy reference database management. *PLoS Comput. Biol.* **17**, e1009581 (2021). 10.1371/journal.pcbi.1009581 PMC8601625 34748542

[43] Gibbons, S. M., Duvallet, C. & Alm, E. J. Correcting for batch effects in case-control microbiome studies. *PLoS Comput. Biol.* **14**, e1006102 (2018). 10.1371/journal.pcbi.1006102 PMC5940237 29684016

