# Supplementary material for: Fecal microbiota transplantation for the decolonization of mucosal adherent-invasive Escherichia coli in Crohn’s disease: an open-label pilot study
Source: Nat Commun. 2026 Sep 10;17:10678. doi: 10.1038/s41467-026-77455-7 (PMC13655058; doi:10.1038/s41467-026-77455-7)
Supplement: Supplementary file 1 [file 41467_2026_77455_MOESM1_ESM.pdf]

Supplementary Information

Fecal Microbiota Transplantation for the Decolonization of Mucosal Adherent-Invasive *Escherichia coli* in Crohn's Disease: An Open-Label Pilot Study

Zhilu XU, Hui JIANG, Wenli HUANG, Alicia CHAN, Yu LIN, Tingting CHAN, Rashid N LUI, Marc T L WONG, Joyce W Y MAK, Francis K L CHAN, Caroline CHEVARIN, Jean-Frédéric COLOMBEL, Anthony BUISSON, Siew C NG

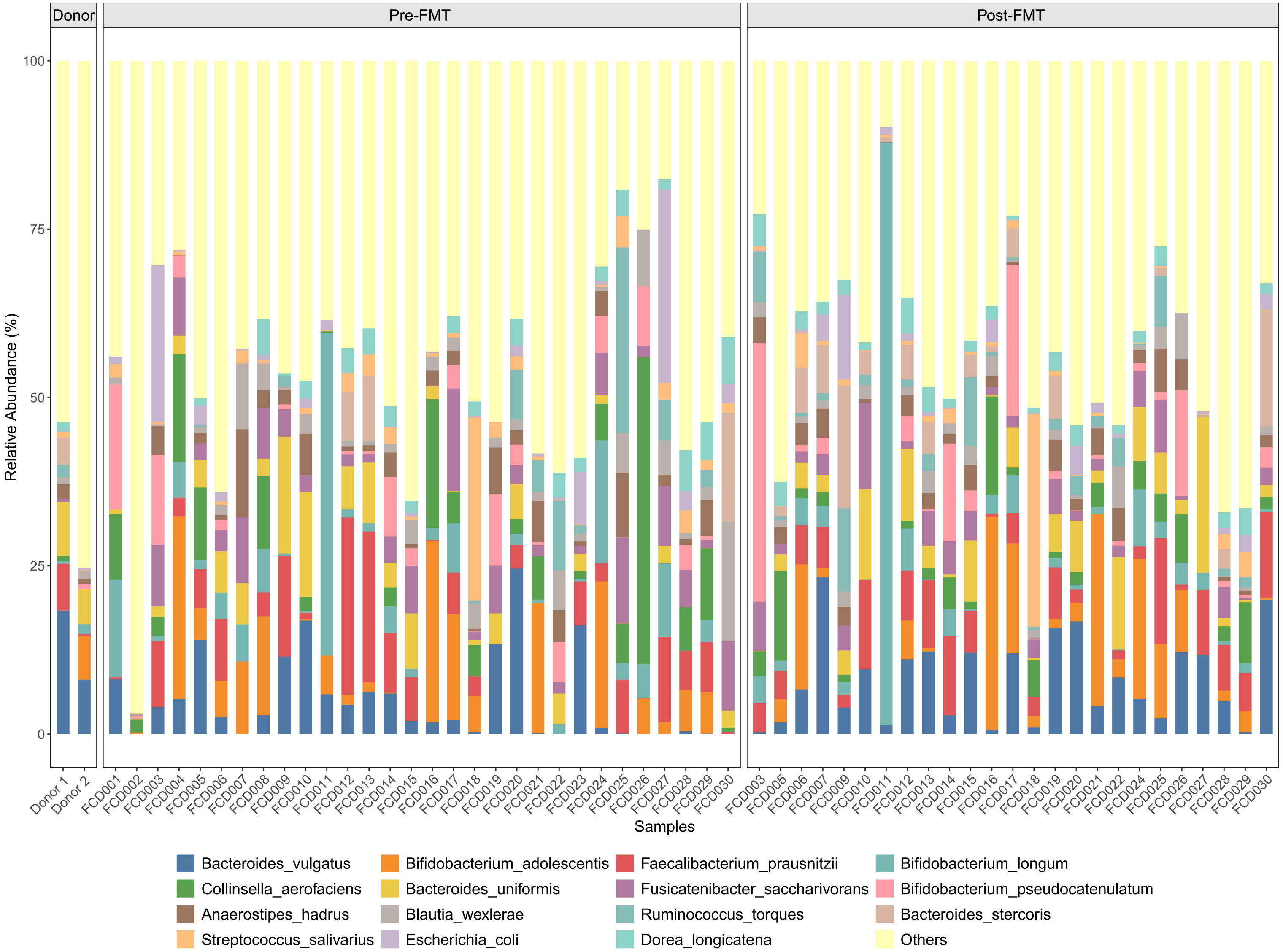

Supplementary Fig. 1 . Species-level composition of the fecal microbiome before and after FMT (related to Fig. 2a). Species-level microbiota composition of fecal samples from donors and from patients before (Pre-FMT, *n*=30 samples) and 12 weeks after FMT (Post-FMT, *n*=25 samples). The 15 most abundant species across all samples are shown, and less abundant species are grouped as Others. Relative abundances are expressed as percentages. FMT, fecal microbiota transplantation.

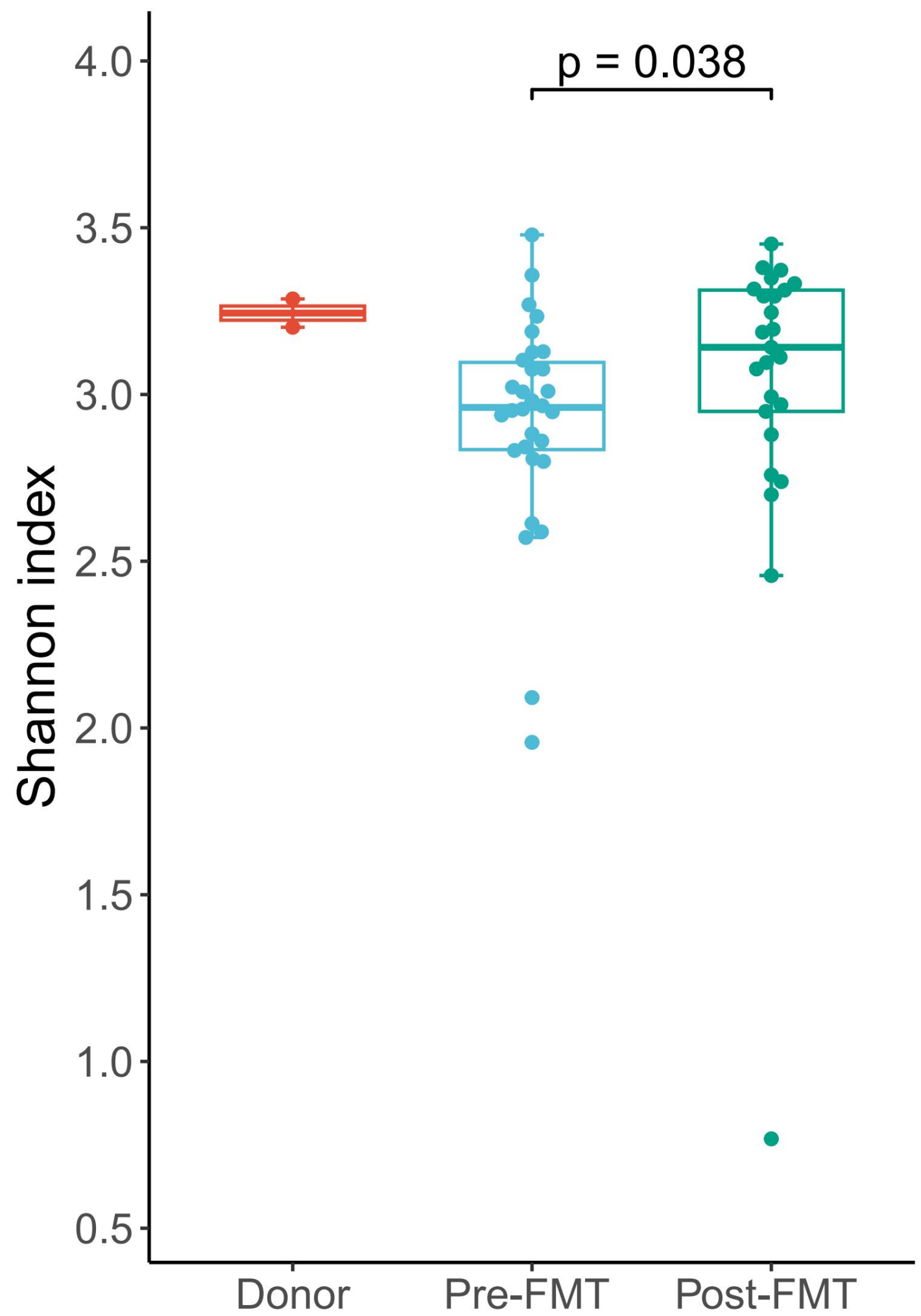

Supplementary Fig. 2 . Fecal alpha diversity before and after FMT using unpaired samples (related to Fig. 2b). Shannon diversity was compared between Pre-FMT (*n*=30) and Post-FMT (*n*=25) samples using a two-sided Wilcoxon rank-sum test. Box plots show the median (centre line), 25th – 75th percentiles (box), and the most extreme values within 1.5 times the interquartile range (whiskers); points represent individual samples. FMT, fecal microbiota transplantation.

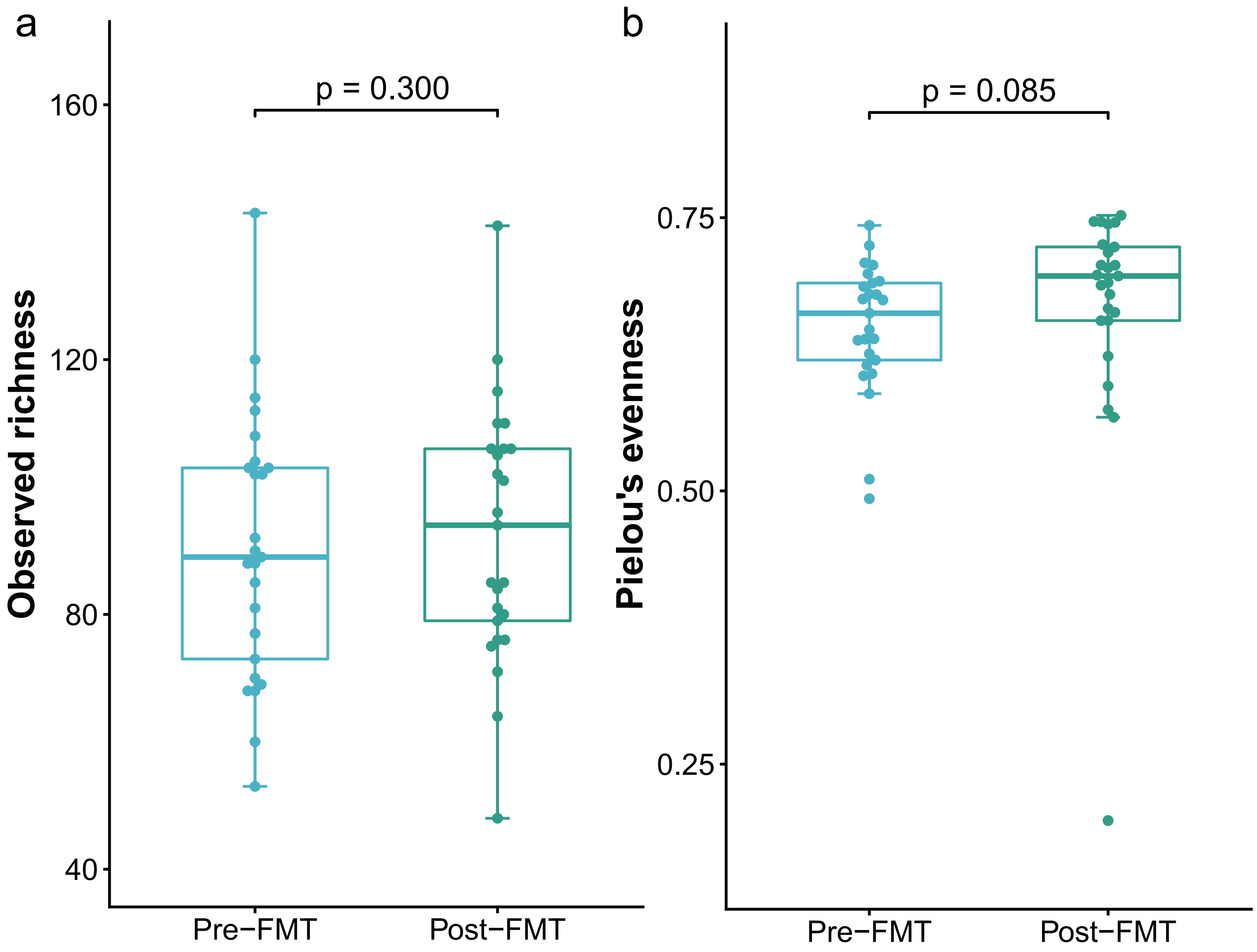

Supplementary Fig. 3. Additional fecal alpha diversity metrics before and after FMT (related to Fig. 2b). (a) Observed richness. (b) Pielou's evenness. Paired Pre-FMT and Post-FMT samples (*n*=25) were compared using two-sided paired Wilcoxon signed-rank tests. Box plots show the median (centre line), 25th – 75th percentiles (box), and the most extreme values within 1.5 times the interquartile range (whiskers); points represent individual participants. FMT, fecal microbiota transplantation.

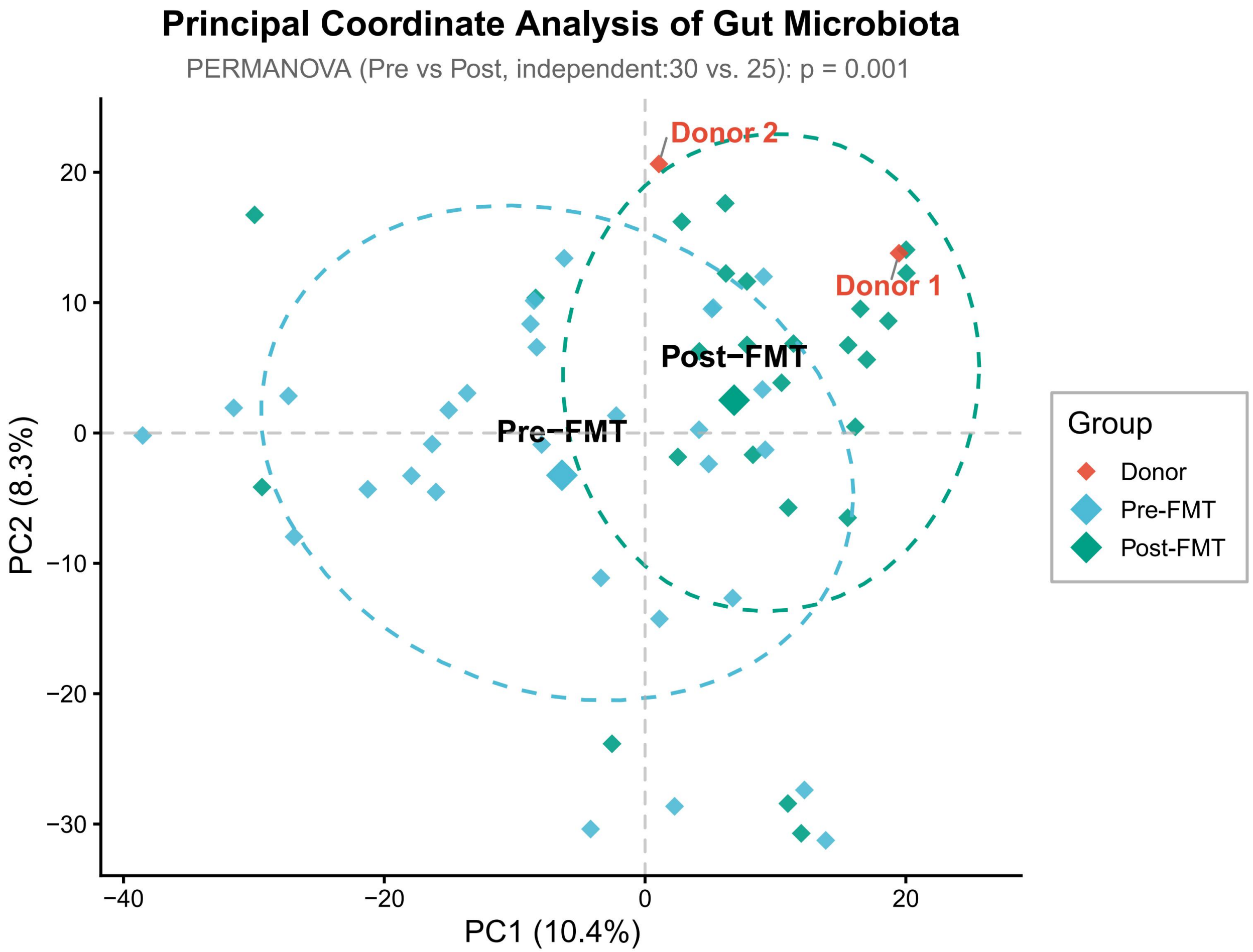

Supplementary Fig. 4. Fecal Beta diversity before and after FMT (unpaired) (related to Fig. 2c). PCoA based on Aitchison distance comparing Pre-FMT (*n*=30) and Post-FMT (*n*=25) fecal samples. Group centroids and confidence ellipses are shown. Differences between timepoints were assessed using PERMANOVA. FMT, fecal microbiota transplantation; PCoA, principal coordinate analysis; PERMANOVA, permutational multivariate analysis of variance.

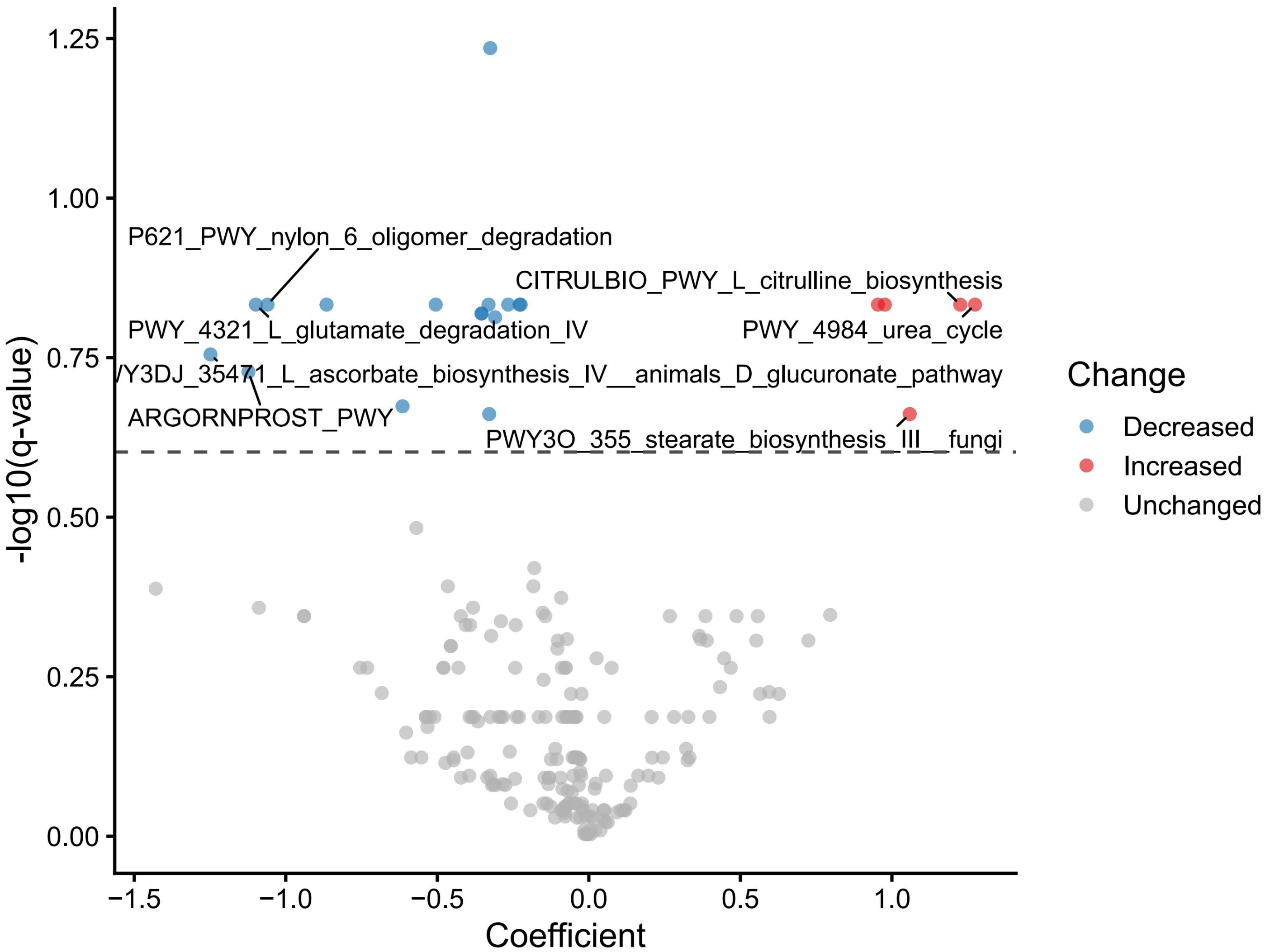

Supplementary Fig. 5. Differentially abundant microbial metabolic pathways after FMT (related to Fig. 2d). MaAsLin2 was applied to paired fecal metagenomic profiles (*n*=25), with subject as a random effect. Red, blue and grey indicate increased, decreased and unchanged pathways, respectively. Pathways with FDR-adjusted *p* value (*q*) ≤0.25 and |coefficient|>1 are labelled.FMT, fecal microbiota transplantation; MaAsLin2, Microbiome Multivariable Association with Linear Models; FDR, false discovery rate.

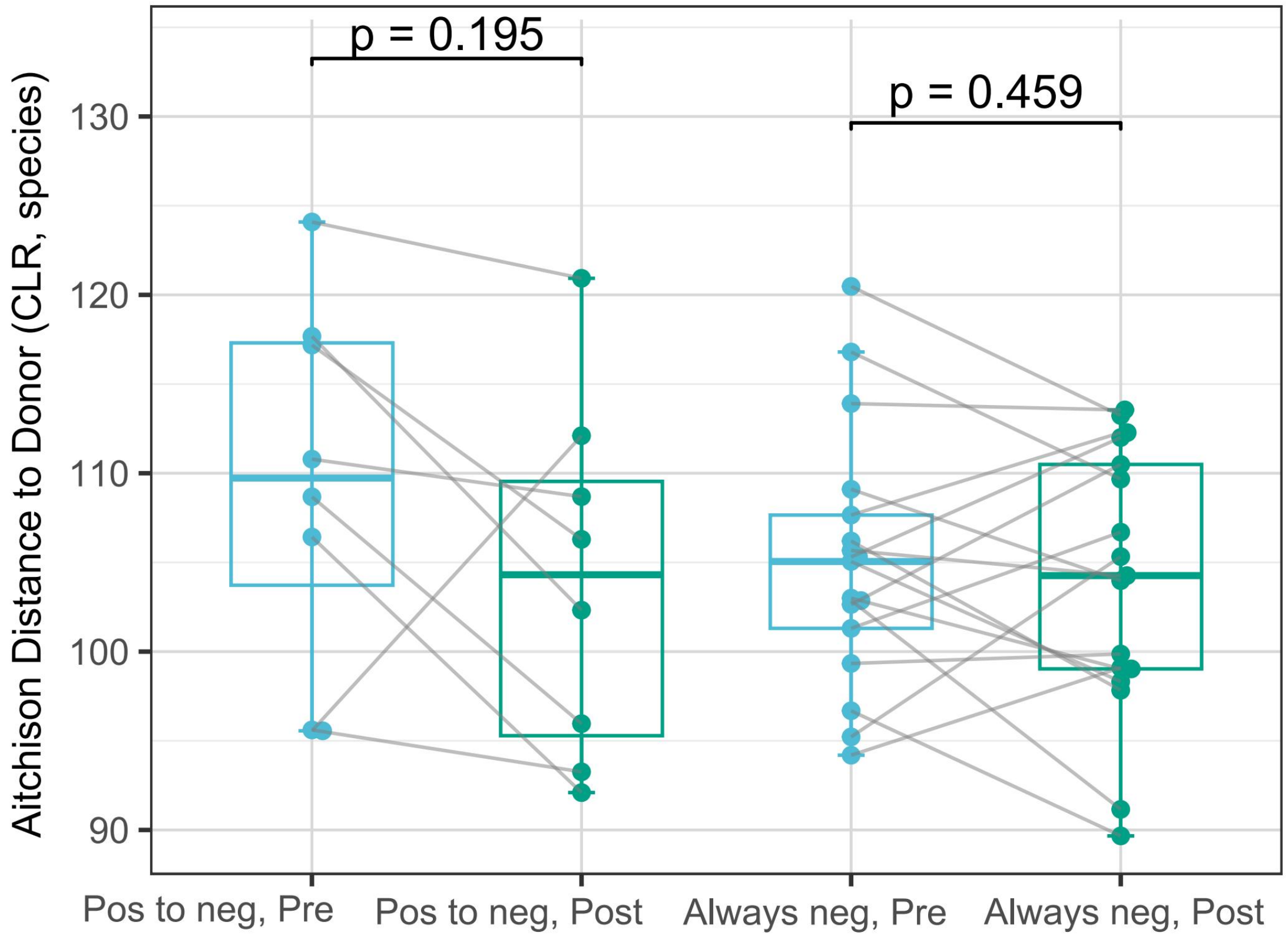

Supplementary Fig. 6. Donor-recipient dissimilarity before and after FMT at the species level, stratified by AIEC status (related to Fig. 2g). Aitchison distance to the corresponding donor was calculated from CLR-transformed species profiles before and after FMT, stratified by AIEC status change (Positive-to-negative, *n*=8 recipients; Always negative, *n*=17 recipients). Grey lines connect paired samples. Within each subgroup, *p* values were calculated using two-sided paired Wilcoxon signed-rank tests. Box plots show the median (centre line), 25th – 75th percentiles (box), and the most extreme values within 1.5 times the interquartile range (whiskers); points represent individual recipients. FMT, fecal microbiota transplantation; AIEC, adherent-invasive *E. coli*; CLR, centered log-ratio.

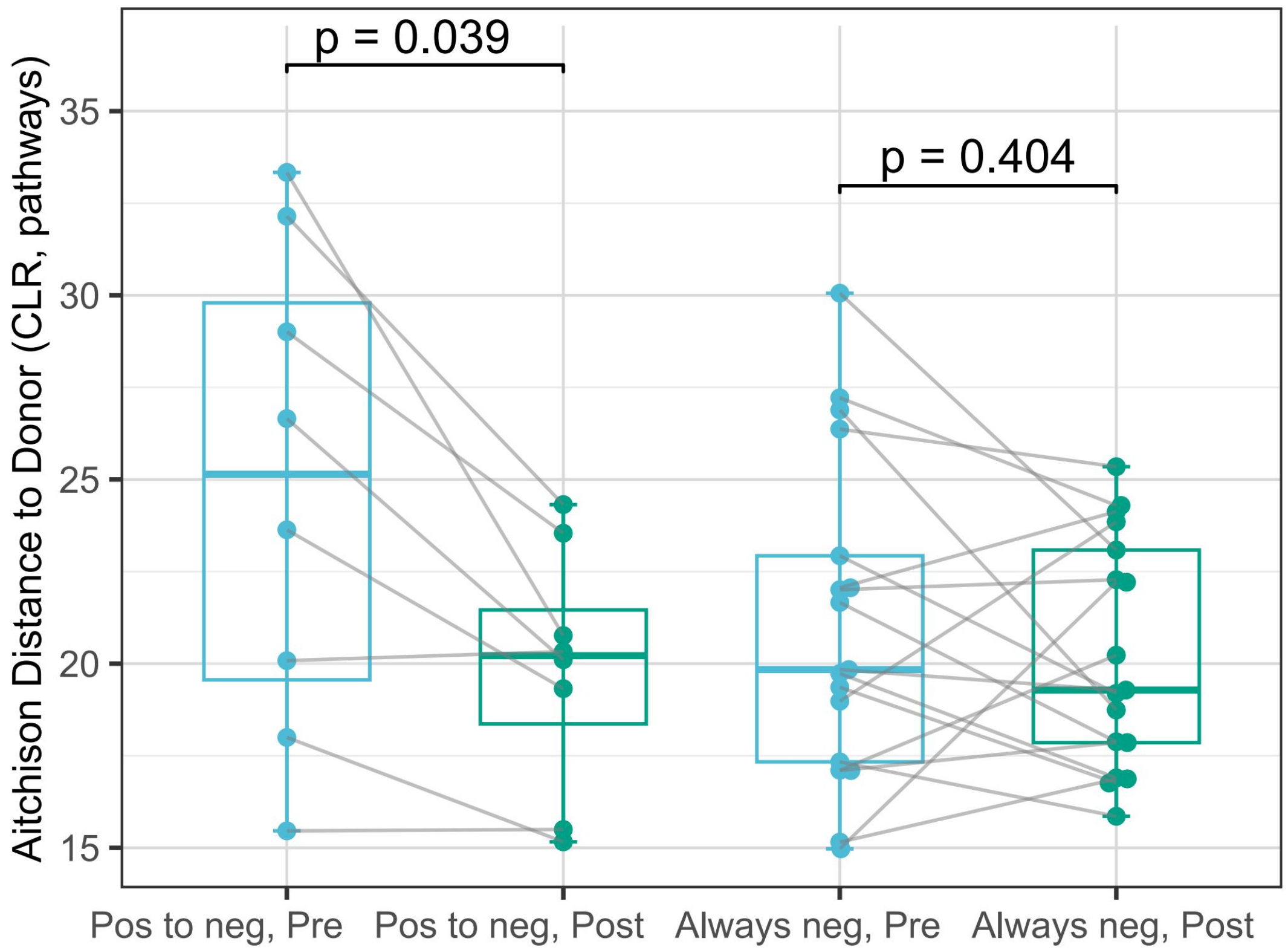

Supplementary Fig. 7. Donor-recipient dissimilarity before and after FMT at the functional level, stratified by AIEC status (related to Fig. 2h). Aitchison distance to the corresponding donor was calculated from CLR-transformed MetaCyc pathway profiles before and after FMT, stratified by AIEC status change (Positive-to-negative, n=8 recipients; Always negative, n=17 recipients). Grey lines connect paired samples. Within each subgroup, p values were calculated using two-sided paired Wilcoxon signed-rank tests. Box plots show the median (centre line), 25th – 75th percentiles (box), and the most extreme values within 1.5 times the interquartile range (whiskers); points represent individual recipients. FMT, fecal microbiota transplantation; AIEC, adherent-invasive *E. coli*; CLR, centered log-ratio.

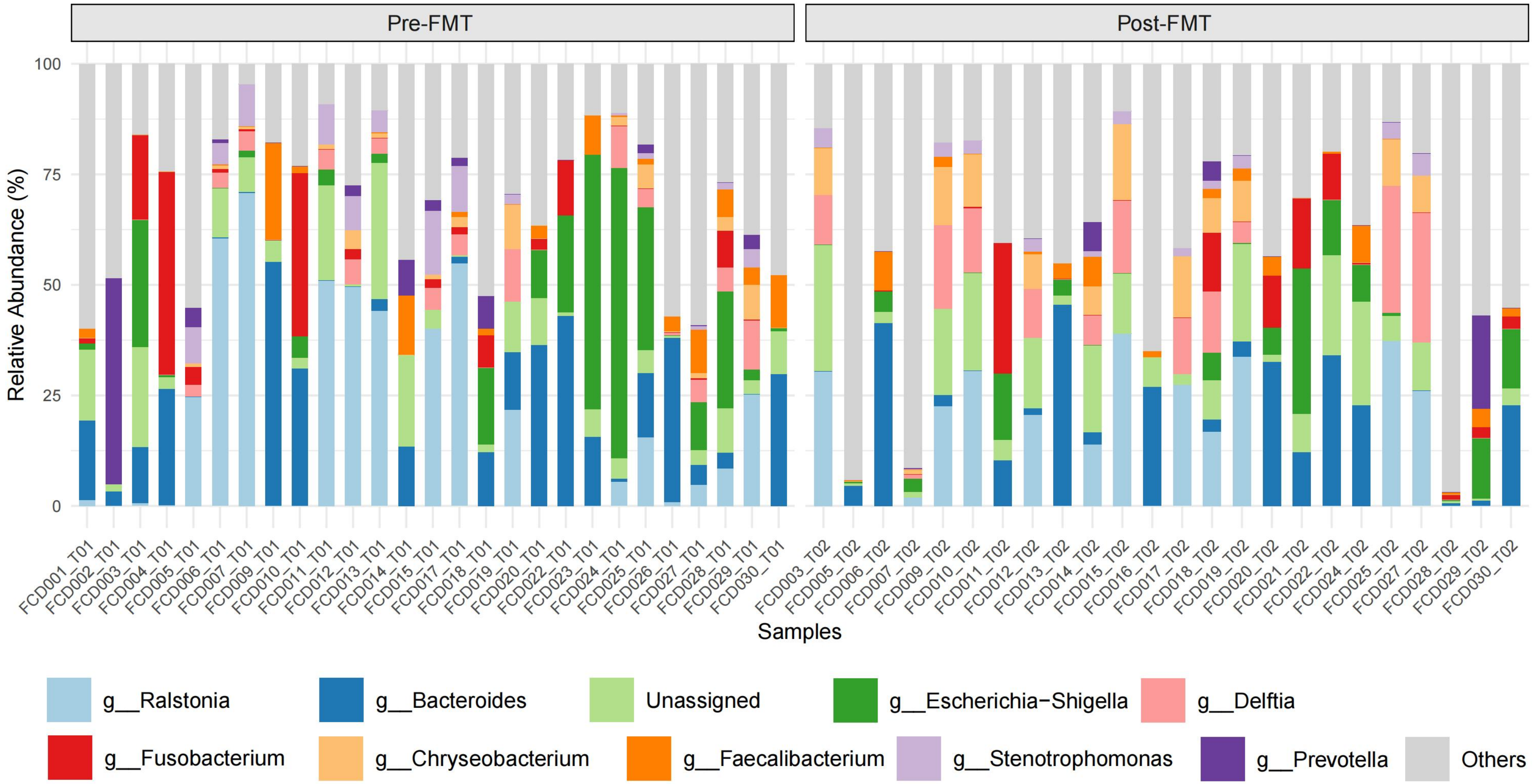

Supplementary Fig. 8. Genus-level composition of the mucosal microbiome before and after FMT (related to Fig. 3a). Genus-level microbiota composition of mucosal biopsy sample scollected before FMT (Pre-FMT, *n*=27 samples) and at follow-up after FMT (Post-FMT, *n*=24 samples).The 10 most abundant genera across all samples are shown, and less abundant genera are grouped as Others. Relative abundances are expressed as percentages.

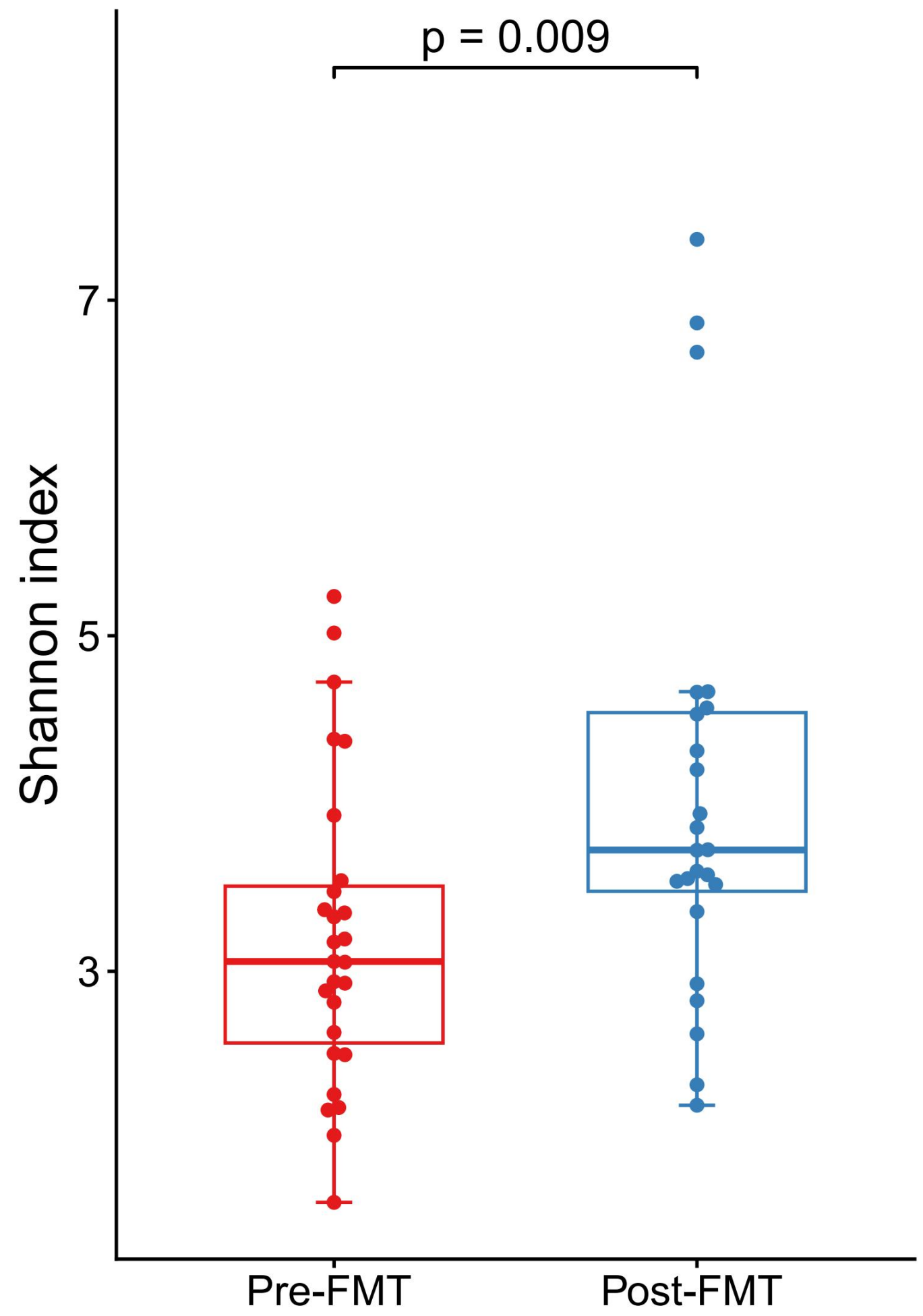

Supplementary Fig. 9 . Mucosal alpha diversity before and after FMT (unpaired) (related to Fig. 3b). Shannon diversity was compared between Pre-FMT (*n*=27) and Post-FMT (*n*=24) mucosal samples using a two-sided Wilcoxon rank-sum test. Box plots show the median (centre line), 25th – 75th percentiles (box), and the most extreme values within 1.5 times the interquartile range (whiskers); points represent individual samples. FMT, fecal microbiota transplantation.

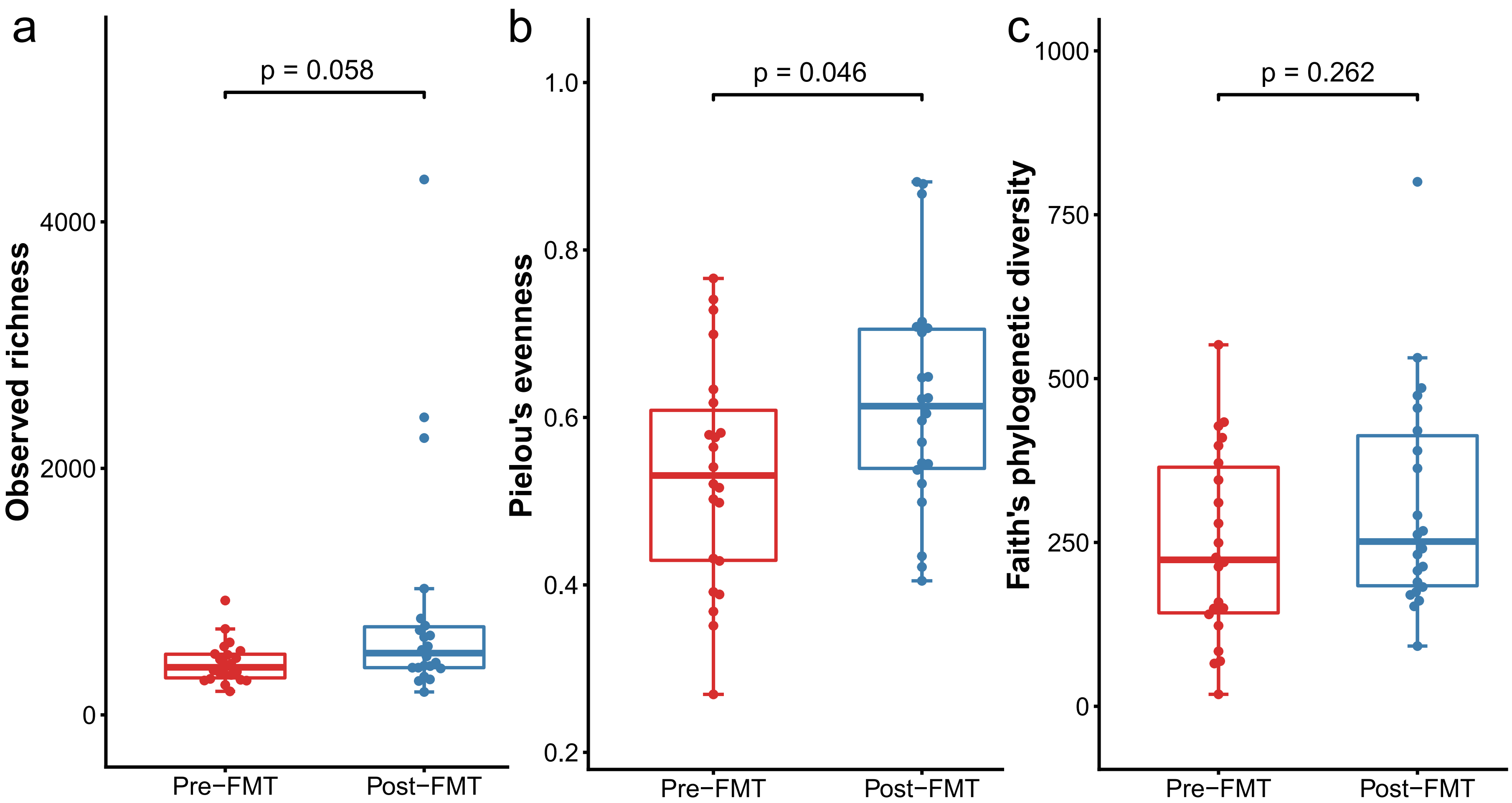

Supplementary Fig. 10. Additional alpha diversity metrics of the mucosal microbiome before and after FMT (related to Fig. 3b). (a) Observed richness. (b) Pielou's evenness. (c) Faith's phylogenetic diversity. Paired Pre-FMT and Post-FMT mucosal samples (*n*=22) were compared using two-sided paired Wilcoxon signed-rank tests. Box plots show the median (centre line), 25th – 75th percentiles (box), and the most extreme values within 1.5 times the interquartile range (whiskers); points represent individual participants. FMT, fecal microbiota transplantation.

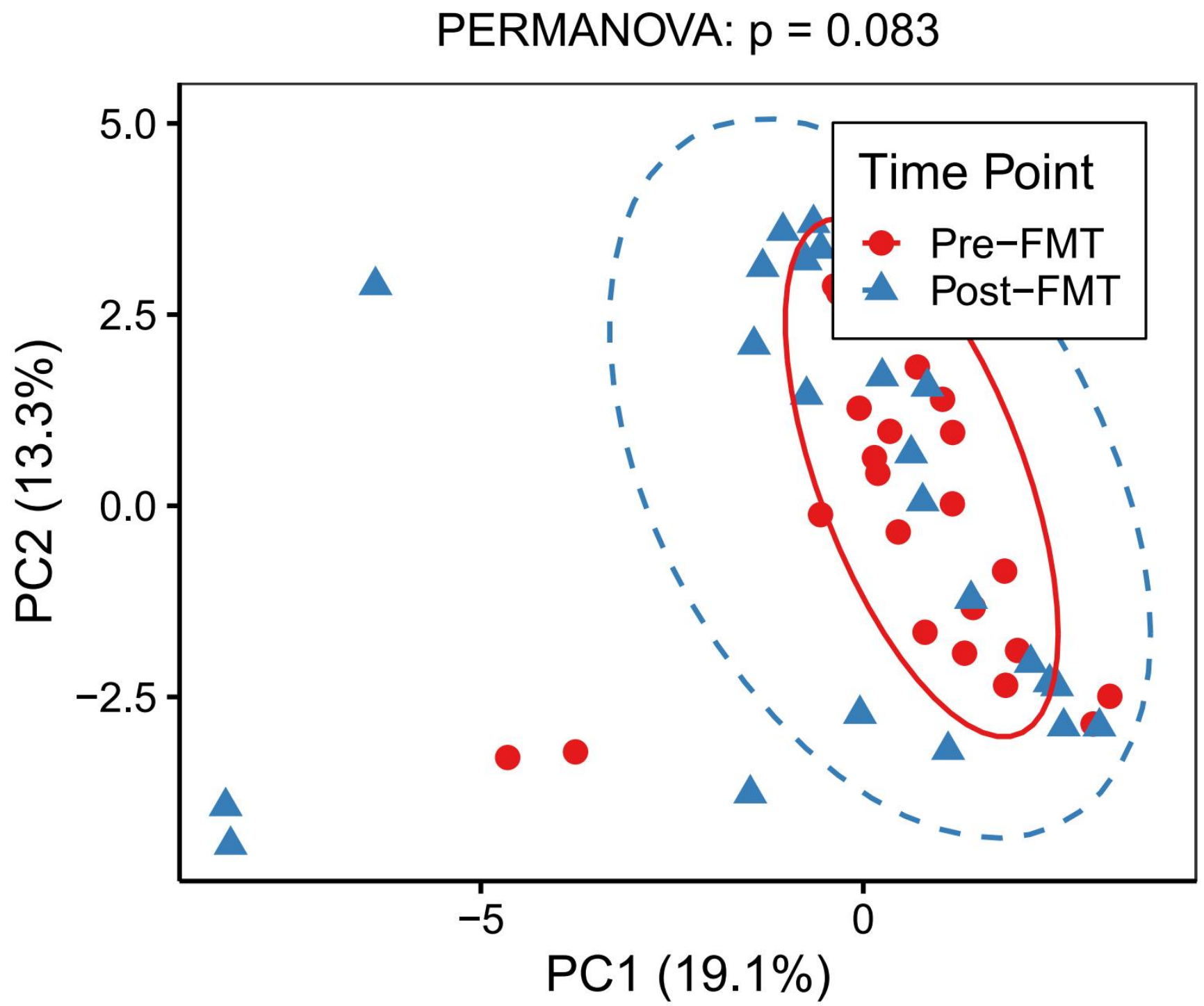

Supplementary Fig. 11. Mucosal beta diversity before and after FMT (unpaired) (related to Fig. 3c). PCoA based on Aitchison distance comparing Pre-FMT (*n*=27) and Post-FMT (*n*=24) mucosal samples. Confidence ellipses are shown. Differences between timepoints were assessed using PERMANOVA. FMT, fecal microbiota transplantation; PCoA, principal coordinate analysis; PERMANOVA, permutational multivariate analysis of variance.

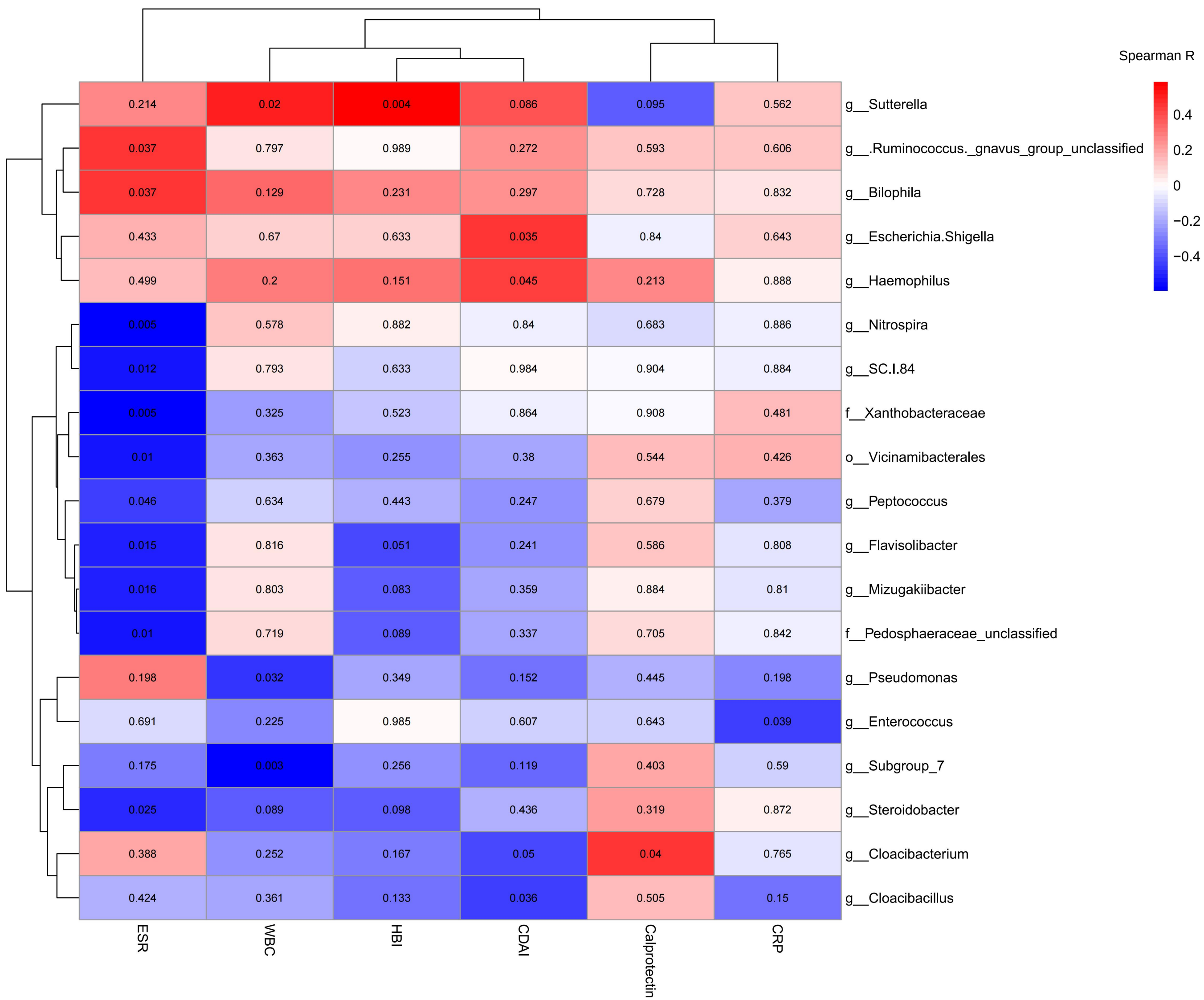

	ESR	WBC	HBI	CDAI	Calprotectin	CRP
g__Sutterella	0.214	0.02	0.004	0.086	0.095	0.562
g__.Ruminococcus._gnavus_group_unclassified	0.037	0.797	0.989	0.272	0.593	0.606
g__Bilophila	0.037	0.129	0.231	0.297	0.728	0.832
g__Escherichia.Shigella	0.433	0.67	0.633	0.035	0.84	0.643
g__Haemophilus	0.499	0.2	0.151	0.045	0.213	0.888
g__Nitrospira	0.005	0.578	0.882	0.84	0.683	0.886
g__SC.I.84	0.012	0.793	0.633	0.984	0.904	0.884
f__Xanthobacteraceae	0.005	0.325	0.523	0.864	0.908	0.481
o__Vicinamibacterales	0.01	0.363	0.255	0.38	0.544	0.426
g__Peptococcus	0.046	0.634	0.443	0.247	0.679	0.379
g__Flavisolibacter	0.015	0.816	0.051	0.241	0.586	0.808
g__Mizugakiibacter	0.016	0.803	0.083	0.359	0.884	0.81
f__Pedosphaeraceae_unclassified	0.01	0.719	0.089	0.337	0.705	0.842
g__Pseudomonas	0.198	0.032	0.349	0.152	0.445	0.198
g__Enterococcus	0.691	0.225	0.985	0.607	0.643	0.039
g__Subgroup_7	0.175	0.003	0.256	0.119	0.403	0.59
g__Steroidobacter	0.025	0.089	0.098	0.436	0.319	0.872
g__Cloacibacterium	0.388	0.252	0.167	0.05	0.04	0.765
g__Cloacibacillus	0.424	0.361	0.133	0.036	0.505	0.15

Supplementary Fig. 12. Associations between changes in selected mucosal genera and clinical/inflammatory indices (related to Fig. 3d). Heatmap shows two-sided Spearman correlations of Post-Pre changes in CLR-transformed mucosal genera with clinical/inflammatory indices. Genera identified by MaAsLin2 as differentially abundant after FMT and *Escherichia-Shigella* were included; only those significantly associated with at least one index are shown. Cell colours indicate Spearman R and numbers indicate p values. HBI, Harvey-Bradshaw Index; CDAI, Crohn's Disease Activity Index; ESR, erythrocyte sedimentation rate; WBC, white blood cell count; CRP, C-reactive protein.

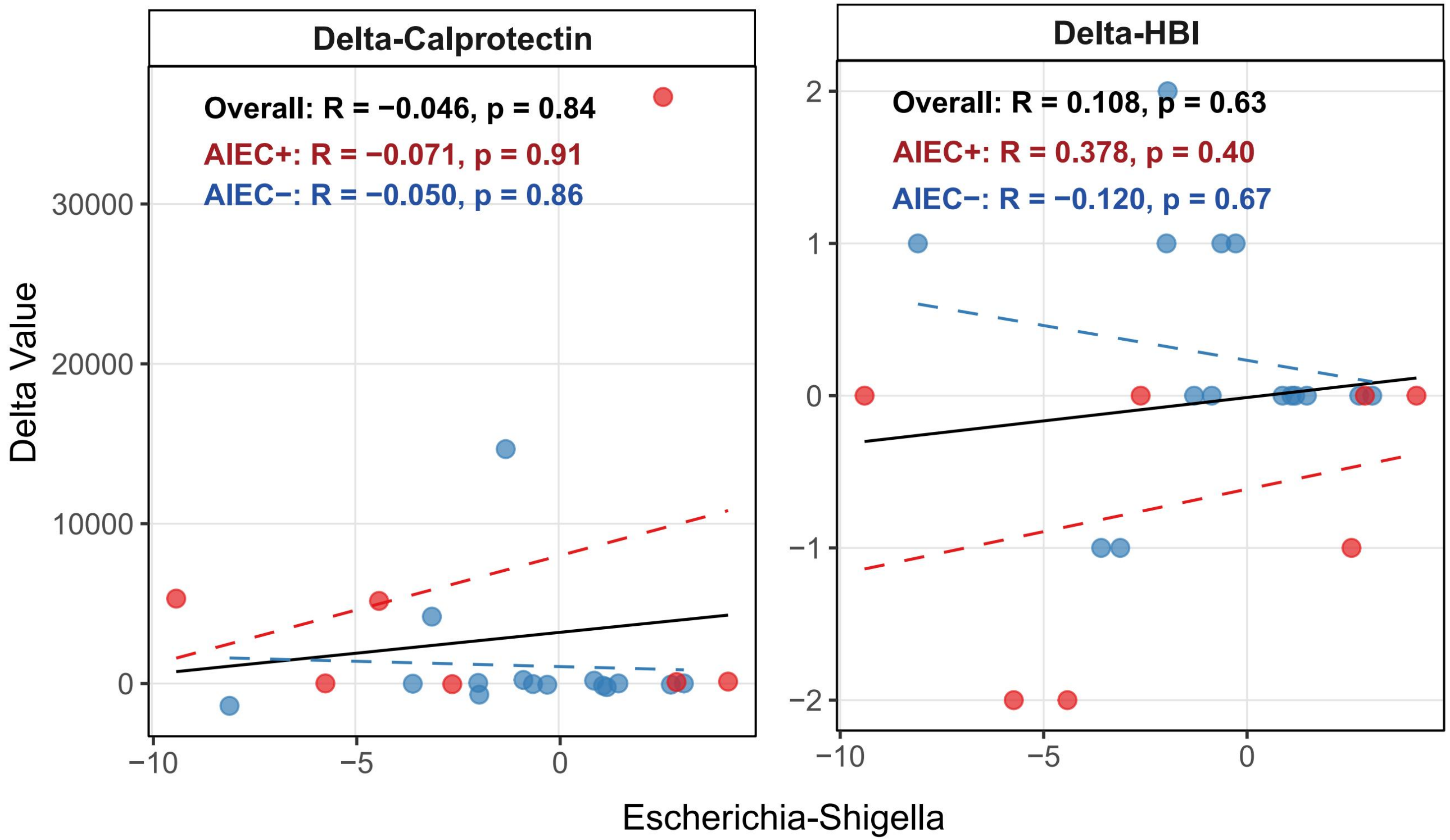

Supplementary Fig. 13. Associations between changes in mucosal *Escherichia-Shigella* and additional clinical/inflammatory indices (related to Fig. 3h). Associations between changes in CLR-transformed mucosal *Escherichia-Shigella* abundance and fecal calprotectin (log-transformed) or HBI (Post-Pre). Solid and dashed lines indicate overall and baseline AIEC subgroup fits, respectively. Two-sided Spearman *R* and *p* values are shown for the overall cohort and each subgroup. CLR, centered log-ratio; HBI, Harvey-Bradshaw Index; AIEC, adherent-invasive *E. coli.*

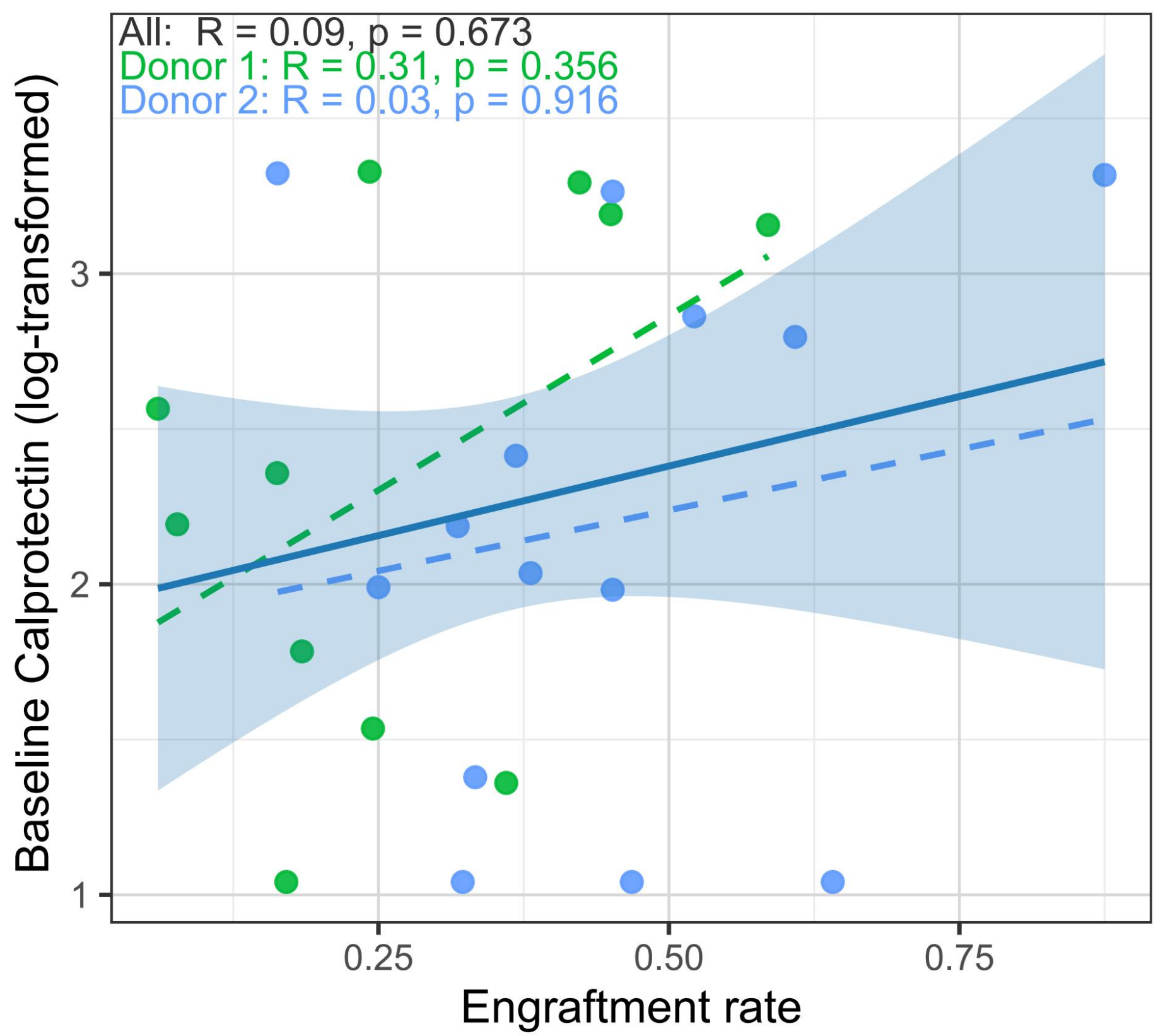

Supplementary Fig. 14. Association between baseline fecal calprotectin and donor microbiota engraftment (related to Fig. 4b). Association between species-level donor engraftment rate and baseline fecal calprotectin (log-transformed). Green and blue indicate Donor 1 and Donor 2, respectively. The solid line and shaded area indicate the overall linear fit and 95% confidence interval; dashed lines indicate donor-specific fits. Two-sided Spearman *R* and *p* values are shown for the overall cohort and each donor group.

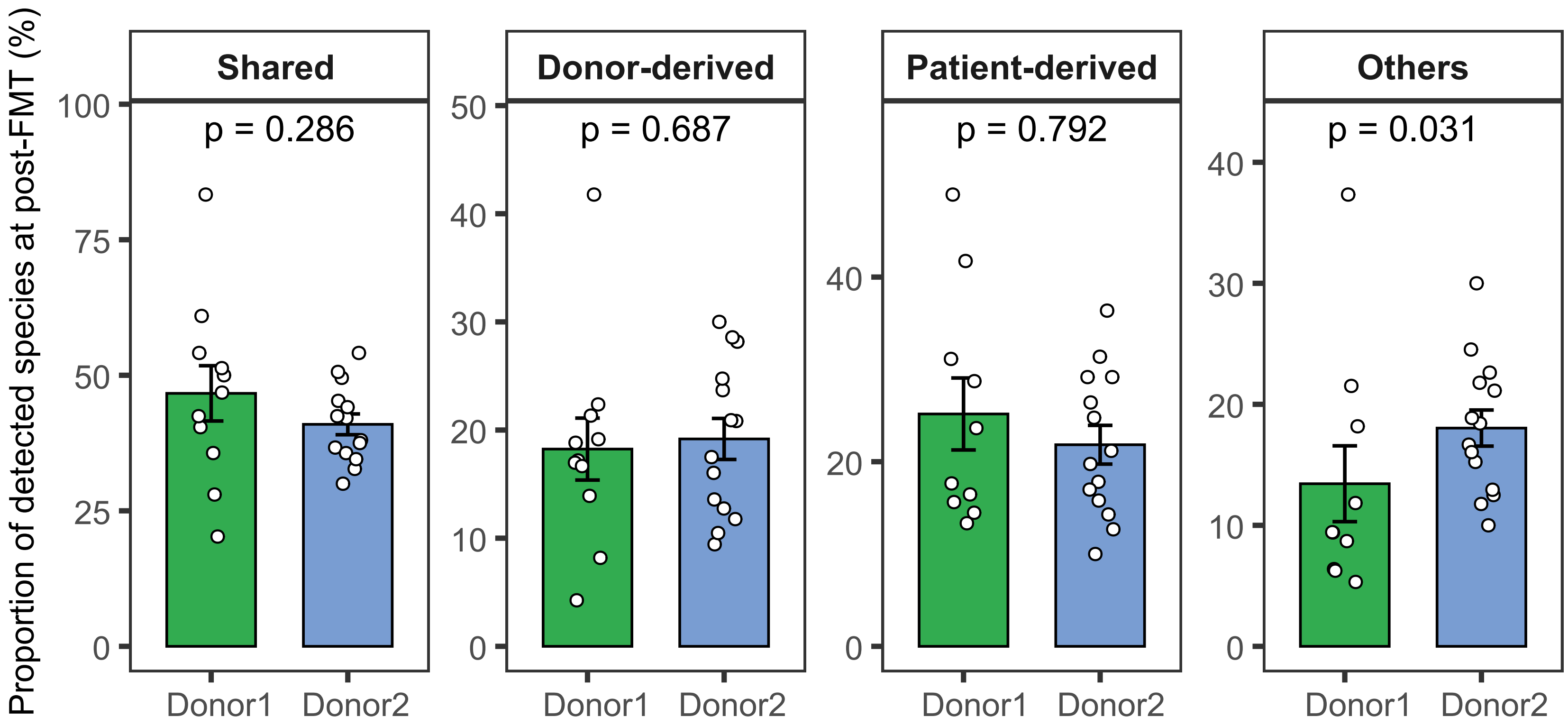

Supplementary Fig. 15. Post-FMT species source composition stratified by donor group (related to Fig. 4h). Proportions of species classified as shared, donor-derived, patient-derived, or others at Week 12 after FMT (*n*=25 recipients), stratified by donor group. Bars show mean proportions, error bars indicate SEM, and overlaid points represent individual recipients. Green and blue indicate Donor 1 and Donor 2, respectively. Donor groups were compared using two-sided Wilcoxon rank-sum tests. FMT, fecal microbiota transplantation; SEM, standard error of the mean.

Supplementary Table 1. Changes in disease activity and laboratory parameters from baseline to week 12 after FMT, stratified by baseline mucosal AIEC status (AIEC positive (*n*=8) vs AIEC negative (*n*=17))

Characteristics	Baseline AIEC colonization (+)				Baseline AIEC colonization (-)				*p* value
	Baseline	Follow-up	Δ	*p* value (paired)	Baseline	Follow-up	Δ	*p* value (paired)	(Δ Between groups)
Disease activity scores									
HBI	0.9 (1.2)	0.2 (0.5)	-0.62 (0.92)	0.095	0.7 (0.9)	0.9 (1.2)	0.24 (0.75)	0.216	0.040
CDAI	54.9 (35.9)	52.2 (34.1)	-2.76 (11.06)	0.503	45.6 (31.2)	61.7 (34.6)	16.08 (28.66)	0.034	0.027
Hematology markers									
Hb (g/dL)	12.9 (1.3)	13.0 (1.2)	0.13 (0.64)	0.598	14.5 (1.1)	14.3 (1.4)	-0.19 (0.63)	0.236	0.272
RBC ($\times 10^{12}$/L)	4.7 (0.8)	4.7 (0.9)	0.04 (0.24)	0.692	5.0 (0.7)	4.9 (0.7)	-0.07 (0.21)	0.198	0.320
WBC ($\times 10^{9}$/L)	7.0 (2.0)	6.2 (2.1)	-0.79 (1.06)	0.073	6.5 (1.6)	5.6 (1.3)	-0.87 (0.91)	0.001	0.851
PLT ($\times 10^{9}$/L)	304.1 (60.2)	296.6 (66.6)	-7.50 (46.46)	0.662	295.8 (73.5)	275.2 (61.7)	-20.59 (36.42)	0.033	0.497
Inflammatory markers									
Fecal Calprotectin (μg/g)	913.2 (899.3)	6730.3 (13309.0)	5817.17 (12714.56)	0.237	516.6 (765)	1501.8 (4120.0)	985.20 (3696.97)	0.288	0.324
CRP (mg/L)	4.0 (6.9)	3.5 (5.3)	-0.45 (1.82)	0.507	2.4 (4.6)	2.3 (5.1)	-0.06 (1.88)	0.889	0.633
ESR (mm/h)	18.4 (8.0)	20.8 (9.0)	2.38 (3.81)	0.122	17.8 (16.7)	16.9 (14.1)	-0.82 (4.45)	0.456	0.083
Biochemical indicators									
Albumin (g/L)	38.1 (5.6)	38.6 (3.6)	0.50 (3.74)	0.717	40.7 (3.2)	39.8 (3.1)	-0.88 (1.65)	0.043	0.346
ALP (U/L)	68.1 (16.0)	61.9 (13.4)	-6.25 (6.65)	0.033	69.9 (17.7)	65.5 (16.3)	-4.41 (7.72)	0.032	0.549
ALT (U/L)	17.1 (4.9)	20.5 (7.7)	3.38 (7.05)	0.218	23.8 (11.8)	26.9 (12.4)	3.06 (10.73)	0.257	0.931
TB (μmol/L)	11.9 (8.9)	14.9 (9.6)	3.00 (2.78)	0.018	9.2 (3.8)	13.6 (4.8)	4.41 (3.76)	0.0002	0.306
Creatinine (μmol/L)	69.4 (10.4)	68.8 (16.3)	-0.63 (8.68)	0.844	76.9 (11.4)	76.1 (10.8)	-0.88 (7.04)	0.613	0.943
Potassium (mmol/L)	3.8 (0.3)	3.7 (0.3)	-0.09 (0.30)	0.443	4.0 (0.3)	3.9 (0.4)	-0.13 (0.45)	0.250	0.787
Sodium (mmol/L)	139.6 (1.9)	139.1 (2.7)	-0.50 (2.88)	0.638	140.4 (1.5)	138.7 (1.8)	-1.71 (1.90)	0.002	0.306

Abbreviations: FMT, fecal microbiota transplantation; AIEC, Adherent-Invasive *E. coli*; HBI, Harvey-Bradshaw Index; CDAI, Crohn's Disease Activity Index; Hb, Hemoglobin; RBC, Red Blood Cell count; WBC, White Blood Cell count; PLT, Platelets; CRP, C-Reactive Protein; ESR, Erythrocyte Sedimentation Rate; ALP, Alkaline Phosphatase; ALT, Alanine Aminotransferase; TB, Total Bilirubin.

Δ= Post-FMT - Pre-FMT. Within-group *p* values were calculated using two-sided paired t-test. Between-group *p* values for Δ were calculated using two-sided independent t-test comparing Δ between baseline AIEC positive and baseline AIEC negative groups.
