# Supplementary material for: Fecal microbiota transplantation for the decolonization of mucosal adherent-invasive Escherichia coli in Crohn’s disease: an open-label pilot study
Source: Nat Commun. 2026 Sep 10;17:10678. doi: 10.1038/s41467-026-77455-7 (PMC13655058; doi:10.1038/s41467-026-77455-7)
Supplement: Supplementary file 2 [file 41467_2026_77455_MOESM2_ESM.pdf]

nature portfolio

Corresponding author(s): Siew C Ng

Last updated by author(s): Aug 16, 2026

Reporting Summary

Nature Portfolio wishes to improve the reproducibility of the work that we publish. This form provides structure for consistency and transparency in reporting. For further information on Nature Portfolio policies, see our Editorial Policies and the Editorial Policy Checklist.

Statistics

For all statistical analyses, confirm that the following items are present in the figure legend, table legend, main text, or Methods section.

n/a	Confirmed	
☐	☒	The exact sample size (*n*) for each experimental group/condition, given as a discrete number and unit of measurement
☐	☒	A statement on whether measurements were taken from distinct samples or whether the same sample was measured repeatedly
☐	☒	The statistical test(s) used AND whether they are one- or two-sided<br>*Only common tests should be described solely by name; describe more complex techniques in the Methods section.*
☐	☒	A description of all covariates tested
☐	☒	A description of any assumptions or corrections, such as tests of normality and adjustment for multiple comparisons
☐	☒	A full description of the statistical parameters including central tendency (e.g. means) or other basic estimates (e.g. regression coefficient) AND variation (e.g. standard deviation) or associated estimates of uncertainty (e.g. confidence intervals)
☐	☒	For null hypothesis testing, the test statistic (e.g. *F*, *t*, *r*) with confidence intervals, effect sizes, degrees of freedom and *P* value noted<br>*Give P values as exact values whenever suitable.*
☒	☐	For Bayesian analysis, information on the choice of priors and Markov chain Monte Carlo settings
☐	☒	For hierarchical and complex designs, identification of the appropriate level for tests and full reporting of outcomes
☐	☒	Estimates of effect sizes (e.g. Cohen's *d*, Pearson's *r*), indicating how they were calculated

Our web collection on statistics for biologists contains articles on many of the points above.

Software and code

Policy information about availability of computer code

Data collection	For full details see Methods; Trimmomatic (v0.39), KneadData (v0.7.2), MetaPhlAn3 (v3.0.9), HUMAnN3 (v3.0.0), QIIME2 (v2024.10), and SILVA 138 were used to process the microbiome sequencing data.
Data analysis	No custom code was generated for this study. All statistical analyses were conducted using R (v4.3.1) and GraphPad Prism (v8.0). Continuous variables are summarized as mean (standard deviation) and categorical variables as n (%). Between-group comparisons were performed using two-sided Welch's t-tests or Mann-Whitney U tests, as appropriate; within-subject changes from baseline to week 12 were assessed using paired t-tests. Categorical outcomes were compared using chi-square or Fisher's exact tests. Microbiome analyses were conducted separately for fecal shotgun metagenomics and ileal mucosal 16S rRNA data. Species-level taxonomic profiling was performed using MetaPhlAn3 (v3.0.9), and functional profiling was conducted using HUMAnN3 (v3.0.0). Resulting abundance tables were processed using the tidyverse (v2.0.0) and phyloseq (v1.24.2) packages. Alpha diversity was assessed using the Shannon index. Beta diversity was evaluated using Aitchison distances derived from centered log-ratio (CLR)-transformed profiles and visualized by principal coordinate analysis (PCoA). Differential abundance analyses of taxa and pathways were performed using general linear model-based Microbiome Multivariable Association with Linear Models (MaAsLin2, v1.24.1). Benjamini-Hochberg false discovery rate (FDR) correction was applied, and features with FDR-adjusted q values <0.25 were considered statistically significant. Associations between microbiome features and clinical or laboratory measures were evaluated using Spearman's rank correlation. The engraftment rate was calculated as Spd/(Spd+Spp), excluding species shared at baseline and species with unknown origin. No covariates were included in the statistical models given the single-arm exploratory design of this pilot study.

For manuscripts utilizing custom algorithms or software that are central to the research but not yet described in published literature, software must be made available to editors and reviewers. We strongly encourage code deposition in a community repository (e.g. GitHub). See the Nature Portfolio guidelines for submitting code & software for further information.

Data

Policy information about availability of data

All manuscripts must include a data availability statement. This statement should provide the following information, where applicable:

- Accession codes, unique identifiers, or web links for publicly available datasets
- A description of any restrictions on data availability
- For clinical datasets or third party data, please ensure that the statement adheres to our policy

The raw sequencing data generated in this study have been deposited in the NCBI Sequence Read Archive (SRA) under BioProject accession codes PRJNA1401743 (https://www.ncbi.nlm.nih.gov/bioproject/?term=PRJNA1401743) and PRJNA1401742 (https://www.ncbi.nlm.nih.gov/bioproject/?term=PRJNA1401742). The full results of the analyses, including all exact p values, and de-identified subject-level clinical metadata are provided in the Source Data file. The clinical metadata can be linked to the corresponding sequencing files.

Research involving human participants, their data, or biological material

Policy information about studies with human participants or human data. See also policy information about sex, gender (identity/presentation), and sexual orientation and race, ethnicity and racism.

Reporting on sex and gender	A total of 30 participants were included in this study, of whom 8 (26.7%) were female (Table 1). Sex (male/female) was self-reported by participants. No sex-disaggregated analysis was performed because of the exploratory nature of this pilot study and because the study was not powered for subgroup analyses by sex.
Reporting on race, ethnicity, or other socially relevant groupings	All the subjects recruited in this study were Hong Kong Chinese.
Population characteristics	This study involved 30 adults with confirmed Crohn's disease (mean age, 35.6 years [SD 12.1]; 8 [26.7%] female) who received a single colonoscopic FMT. Additional demographic and clinical characteristics are provided in Table 1.
Recruitment	Patients were included if they met the following criteria: (1) age >18 years; (2) confirmed diagnosis of Crohn's disease (CD) with a disease duration ≥3 months; (3) no or mild-to-moderate symptoms, defined as a Harvey-Bradshaw Index (HBI) score <16; and (4) seropositivity for AEcAb. Exclusion criteria included pregnancy; initiation of new biologic therapy or corticosteroid use within 4 weeks prior to enrollment; presence of colorectal tumor; active gastrointestinal bleeding; diagnosis of ulcerative colitis; history of colectomy or partial colectomy with ileo-transverse colonic anastomosis; presence of colonic or small bowel stoma; active perianal lesions; antibiotic use within 4 weeks prior to enrollment; and inability to provide informed consent due to psychological or linguistic incapacity. Participants did not receive compensation for participation in the study.
Ethics oversight	The study was approved by the Joint Chinese University of Hong Kong of New Territories East Cluster Clinical Research Ethics Committee (CUHK-NTEC CREC). All subjects provided written informed consent.

Note that full information on the approval of the study protocol must also be provided in the manuscript.

Field-specific reporting

Please select the one below that is the best fit for your research. If you are not sure, read the appropriate sections before making your selection.

[x] Life sciences [ ] Behavioural & social sciences [ ] Ecological, evolutionary & environmental sciences

For a reference copy of the document with all sections, see nature.com/documents/nr-reporting-summary-flat.pdf

Life sciences study design

All studies must disclose on these points even when the disclosure is negative.

Sample size	This pilot study enrolled a total of 30 participants. The sample size was determined based on feasibility and the exploratory nature of the study, and no formal sample size calculation was performed.
Data exclusions	No data were excluded from the analyses; sample availability for individual analyses is reported in the manuscript and figure legends.
Replication	No independent replication cohort was included in this pilot clinical study. Standardized procedures and analytical pipelines were used to support reproducibility.
Randomization	No randomization.
Blinding	Blinding to group allocation was not applicable because this was a single-arm, open-label study in which all participants received FMT.

Reporting for specific materials, systems and methods

We require information from authors about some types of materials, experimental systems and methods used in many studies. Here, indicate whether each material, system or method listed is relevant to your study. If you are not sure if a list item applies to your research, read the appropriate section before selecting a response.

Materials & experimental systems

n/a	Involved in the study
☐	☒ Antibodies
☐	☒ Eukaryotic cell lines
☒	☐ Palaeontology and archaeology
☒	☐ Animals and other organisms
☐	☒ Clinical data
☒	☐ Dual use research of concern
☒	☐ Plants

Methods

n/a	Involved in the study
☒	☐ ChIP-seq
☒	☐ Flow cytometry
☒	☐ MRI-based neuroimaging

Antibodies

Antibodies used	HRP-conjugated goat anti-human IgG secondary antibody (Sigma-Aldrich; dilution 1:50,000)
Validation	Not applicable; no primary antibodies were used in this study.

Eukaryotic cell lines

Policy information about cell lines and Sex and Gender in Research

Cell line source(s)	Intestine-407 cells (ATCC CCL-6), obtained from the American Type Culture Collection (ATCC)
Authentication	The cell line was obtained from ATCC (CCL-6). No additional authentication was performed in our laboratory.
Mycoplasma contamination	No additional mycoplasma testing was performed in our laboratory.
Commonly misidentified lines (See ICLAC register)	Intestine-407 (ATCC CCL-6) is a known HeLa-derived cell line. It was used in this study as a standardized cell-based model for assessment of bacterial invasion.

Clinical data

Policy information about clinical studies

All manuscripts should comply with the ICMJE guidelines for publication of clinical research and a completed CONSORT checklist must be included with all submissions.

Clinical trial registration	NCT05611866
Study protocol	Study protocol was submitted as supplementary material. All participants provided written informed consent prior to any study procedures.
Data collection	Participants were recruited and study data were collected between October 2022 and January 2023 at The Chinese University of Hong Kong (CUHK), Hong Kong. Clinical assessments and sample collection, including blood, stool, and ileal mucosal biopsies, were conducted at CUHK.
Outcomes	The primary outcome was the occurrence of adverse events during and within 12 weeks after FMT, assessed and graded using CTCAE v4.03. Secondary outcomes included changes in mucosal AIEC colonization assessed from ileal biopsies, clinical and laboratory measures, and fecal and mucosal microbiome profiles assessed by shotgun metagenomic and 16S rRNA sequencing, respectively.

Plants

Seed stocks	*Report on the source of all seed stocks or other plant material used. If applicable, state the seed stock centre and catalogue number. If plant specimens were collected from the field, describe the collection location, date and sampling procedures.*
Novel plant genotypes	*Describe the methods by which all novel plant genotypes were produced. This includes those generated by transgenic approaches, gene editing, chemical/radiation-based mutagenesis and hybridization. For transgenic lines, describe the transformation method, the number of independent lines analyzed and the generation upon which experiments were performed. For gene-edited lines, describe the editor used, the endogenous sequence targeted for editing, the targeting guide RNA sequence (if applicable) and how the editor was applied.*
Authentication	*Describe any authentication procedures for each seed stock used or novel genotype generated. Describe any experiments used to assess the effect of a mutation and, where applicable, how potential secondary effects (e.g. second site T-DNA insertions, mosiacism, off-target gene editing) were examined.*
