# Supplementary material for: Fecal microbiota transplantation for the decolonization of mucosal adherent-invasive Escherichia coli in Crohn’s disease: an open-label pilot study
Source: Nat Commun. 2026 Sep 10;17:10678. doi: 10.1038/s41467-026-77455-7 (PMC13655058; doi:10.1038/s41467-026-77455-7)
Supplement: Supplementary file 3 [file 41467_2026_77455_MOESM3_ESM.pdf]

nature portfolio

Peer Review File

Fecal Microbiota Transplantation for the Decolonization of Mucosal Adherent-Invasive Escherichia coli in Crohn's Disease: An Open-Label Pilot Study

Corresponding Author: Professor Siew C. Ng

This file contains all reviewer reports in order by version, followed by all author rebuttals in order by version.

Version 0:

Reviewer comments:

Reviewer #1

(Remarks to the Author)
In this study, the authors evaluated the effect of FMT to decolonize AIEC bacteria in patients with Crohn's disease.
A single FMT was performed via colonoscopy in 30 patients with no or mild-to-moderate symptoms. The authors found some interesting results regarding the decolonization of AIEC and the effect on the microbiome. However, the study has several important limitations.

Major comments:

- Evaluation of decolonization at week 12 seems very early. What are the results at a later time point? Moreover, some studies show that carriage of AIEC is transient. What is the AIEC status post FMT in patients who were AIEC- before FMT?

- The authors should show clearly the effect of FMT on disease activity markers (HBI, CDAI, CRP, fecal calprotectin, endoscopic score such as SES-CD) in the whole population and in the AIEC + vs – groups.

- There are multiple statistical tests in the paper. Is there any adjustment for FDR?

- AIEC is associated wilth ileal CD. Why patients with pure colonic lesions were enrolled?

- The authors should globally decrease the tone of their affirmation. There is no control group, the number of subjets is low, it is not possible to make any link between AIEC decolonization and therapeutic effect in this study as patients were in remission.

Minor comments
- where the fecal infusion was performed? in the colon?

Reviewer #2

(Remarks to the Author)

Xu et al. used FMT as a treatment in an attempt to safely clear adherent-invasive E. coli in Crohns disease. The study does have some very nice aspects - such as inclusion of mucosal sampling and the phenotyping of the isolated E. coli. The results are potentially important, however the paper suffers from the issue that there is no placebo and it is not possible to frame clearance of the AIEC as being due to the treatment. Moreover, the sample size means that the sample is very underpowered. This is compounded by the authors inconsistent application of pvalue significance and FDR cut-off using terms like 'trending' or moving towards significance.

This permeates the paper from Table 1 - comparisons of unbalanced sample sets (9 vs 21) by Student's T-test require that

the samples are homoscedastic but this is often violated with sample sample numbers (eg. 9). The authors did not test for violation of this. By contras, Welch's T-test does not assume equal variance, and is robust to unequal sample sizes.

Metaphlan analysis: significant results and results 'trending' to significance are discussed but there is no discussion of the effect size. Only calculated Shannon index, without also calculating the Peilou's evenness, richness and Faith's measures. Differences between these would have enabled greater insight into the structure of the population changes that were happening.

How did the authors confirm that the AIEC were cleared from the samples and did not just drop below the level of detection of the method they used?

With respect to the inconsistent use of p-value cut-offs, the authors selectively report results that help build their story using 'trends' (e.g. "However, among recipients,
377 higher species-level engraftment tended to correlate with greater reductions in fecal
378 calprotectin (log-transformed Δcalprotectin, Spearman's test, p=0.059;" "AEcAb levels remained numerically higher in the
275 baseline AIEC-positive participants but did not reach statistical significance (p=0.076;")

Is the use of a cohort with mild-to-moderate symptoms appropriate for testing clearance? This is mentioned in the discussion yet the paper over-reaches conclusions about "ability of FMT to target key pathobionts".

Causal language is an issue, as study design limits the work to show assoications/correlations.

Limitations should be expanded upon in the discussion.

Open Access This Peer Review File is licensed under a Creative Commons Attribution 4.0 International License, which permits use, sharing, adaptation, distribution and reproduction in any medium or format, as long as you give appropriate credit to the original author(s) and the source, provide a link to the Creative Commons license, and indicate if changes were made.
In cases where reviewers are anonymous, credit should be given to 'Anonymous Referee' and the source.
The images or other third party material in this Peer Review File are included in the article's Creative Commons license, unless indicated otherwise in a credit line to the material. If material is not included in the article's Creative Commons license and your intended use is not permitted by statutory regulation or exceeds the permitted use, you will need to obtain permission directly from the copyright holder.
To view a copy of this license, visit https://creativecommons.org/licenses/by/4.0/

Reviewer(s)' Comments to Author:

Reviewer #1

In this study, the authors evaluated the effect of FMT to decolonize AIEC bacteria in patients with Crohn's disease. A single FMT was performed via colonoscopy in 30 patients with no or mild-to-moderate symptoms. The authors found some interesting results regarding the decolonization of AIEC and the effect on the microbiome. However, the study has several important limitations.

Response: We thank the reviewer for their thorough evaluation of our manuscript and for highlighting important limitations of the study. We have carefully considered these comments and revised the manuscript to clarify the study design, its limitations, and the interpretation of our findings.

Major comments:

1. Evaluation of decolonization at week 12 seems very early. What are the results at a later time point? Moreover, some studies show that carriage of AIEC is transient. What is the AIEC status post FMT in patients who were AIEC- before FMT?

   Response: We thank the reviewer for this important and insightful comment. The week 12 endpoint was selected based on prior FMT studies in Crohn's disease, which commonly assess short-term efficacy within an 8–12 week window following FMT administration (PMID: 38501667; PMID: 28214091). We agree that longer-term follow-up would be valuable to determine the durability of AIEC decolonization. However, assessment of AIEC status requires colonoscopy with mucosal biopsies, an invasive procedure that is not routinely justified at multiple time points in clinically stable patients with no or mild-to-moderate symptoms. We have now acknowledged this as a limitation in the revised manuscript (page 10, lines 338-339). Regarding baseline AIEC-negative patients, all remained AIEC-negative at week 12. This clarification has been added to the Results section (page 4, lines 122-123).

2. The authors should show clearly the effect of FMT on disease activity markers (HBI, CDAI, CRP, fecal calprotectin, endoscopic score such as SES-CD) in the whole population and in the AIEC + vs – groups.

   Response: We thank the reviewer for this valuable suggestion. Changes in clinical disease activity indices (HBI, CDAI) and inflammatory markers (CRP, fecal calprotectin), analyzed both in the overall cohort and stratified by baseline AIEC status, were originally presented in Supplementary Tables 1 and 2. To improve clarity and emphasize these clinically relevant outcomes, we have moved the former Supplementary Table 1 into the main manuscript as Table 2. The stratified analyses by baseline AIEC status are now provided as Supplementary Table S1 (renumbered).

   In summary, FMT did not significantly modify clinical or biochemical parameters over the 12-week follow-up in either AIEC-positive or AIEC-negative patients. This is

consistent with the study population, which comprised individuals with no or mild-to-moderate disease activity at baseline. Endoscopic severity (SES-CD) was not collected in this study, as previously acknowledged (page 10, lines 332-333). We agree that incorporation of endoscopic endpoints in future studies will be important to more directly assess the relationship between AIEC decolonization and mucosal healing.

3. There are multiple statistical tests in the paper. Is there any adjustment for FDR?
Response: We thank the reviewer for raising this important point. To ensure clarity and transparency, we have revised the Methods and Results sections (page 14, lines 497-519) to explicitly describe our approach to multiple-testing correction. For differential abundance analyses of microbial taxa and metabolic pathways, false discovery rate (FDR) correction was applied using the Benjamini–Hochberg procedure as implemented in MaAsLin2. Statistical significance was determined based on FDR-adjusted q values. This has been clarified in the text, figure legends (Figure 2d and Figure S4), and corresponding source data files. For analyses examining associations between microbiome features and clinical or laboratory parameters using Spearman's rank correlation, these were conducted as exploratory assessments. Accordingly, nominal p values are reported without additional multiple-testing correction. Given the exploratory nature of the study—designed to investigate the potential impact of FMT on AIEC decolonization and microbiome dynamics rather than to draw definitive clinical inferences—we believe this approach is appropriate.

4. AIEC is associated with ileal CD. Why patients with pure colonic lesions were enrolled?
Response: We thank the reviewer for this thoughtful and important question. While AIEC has been most strongly associated with ileal Crohn's disease, its presence is not restricted to ileal involvement (PMID: 15300573). Experimental and clinical studies have demonstrated that AIEC can adhere to and colonize colonic epithelium via CEACAM6, which is constitutively expressed on colonic epithelial cells and can be further upregulated under pro-inflammatory conditions (PMID: 17525800; PMID: 40624731; PMID: 29141957; PMID: 40473402). These findings support the biological plausibility of AIEC colonization in colonic disease. Consistent with this literature, 2 of 9 patients with isolated colonic Crohn's disease in our cohort were AIEC-positive at baseline (Table 1), confirming that AIEC colonization can occur in colonic CD, albeit at a lower prevalence than in ileal disease. Furthermore, FMT exerts global effects on the intestinal microbiome rather than being anatomically restricted (PMID: 40482640). Given the exploratory nature of our study and our focus on AIEC decolonization irrespective of disease location, inclusion of patients with colonic involvement does not confound the assessment. Importantly, AIEC was detectable in this subgroup, allowing evaluation of FMT-associated changes in these individuals as well.

5. The authors should globally decrease the tone of their affirmation. There is no control

group, the number of subjets is low, it is not possible to make any link between AIEC decolonization and therapeutic effect in this study as patients were in remission.

Response: We thank the reviewer for this important and constructive comment. We fully agree that the exploratory design of our study including the absence of a control group, the limited sample size, and the inclusion of patients in remission does not allow causal inferences regarding a therapeutic effect of AIEC decolonization. In response, we have carefully revised the manuscript to moderate the tone and avoid any statements that could be interpreted as implying causality or clinical efficacy. The title has been amended to: *"Fecal Microbiota Transplantation for the Decolonization of Mucosal Adherent-Invasive Escherichia coli in Crohn's Disease: An Open-Label Pilot Study"*, to more accurately reflect the scope and design of the work. Additionally, the Abstract, Introduction, Results, Discussion, and Conclusion sections have been systematically revised to emphasize the exploratory, proof-of-concept nature of the study. We now consistently frame our findings as preliminary and hypothesis-generating rather than definitive. The relevant revisions are indicated in the marked manuscript (such as page 1, lines 1-3; page 2, lines 40-54). We have also strengthened the Limitations section (pages 9-10, lines 325-341) to explicitly state that this study was not designed to evaluate clinical efficacy, and that larger, controlled trials will be required to determine whether AIEC decolonization translates into meaningful therapeutic benefit.

Minor comments

1. where the fecal infusion was performed? in the colon?

   Response: FMT was delivered via colonoscopy into the terminal ileum, ensuring that the transplanted material reached the intended site. This detail has been clearly stated in the Methods section (page 11, lines 401-403).

Reviewer #2

1. Xu et al. used FMT as a treatment in an attempt to safely clear adherent-invasive E. coli in Crohns disease. The study does have some very nice aspects - such as inclusion of mucosal sampling and the phenotyping of the isolated E. coli. The results are potentially important, however the paper suffers from the issue that there is no placebo and it is not possible to frame clearance of the AIEC as being due to the treatment. Moreover, the sample size means that the sample is very underpowered. This is compounded by the authors inconsistent application of pvalue significance and FDR cut-off using terms like 'trending' or moving towards significance.

   Response: We thank the reviewer for the thoughtful evaluation of our work and for recognizing the strengths of the study design, including mucosal sampling and detailed phenotyping of isolated *E. coli* strains. We fully acknowledge that the absence of a placebo-controlled arm limits our ability to draw causal conclusions regarding the effect of FMT on AIEC clearance. Accordingly, we have revised the manuscript throughout to avoid causal language and to more clearly frame the findings as

preliminary and hypothesis-generating. The title has been amended to: *"Fecal Microbiota Transplantation for the Decolonization of Mucosal Adherent-Invasive Escherichia coli in Crohn's Disease: An Open-Label Pilot Study."* The Abstract, Introduction, Results, Discussion, and Conclusions have been carefully revised to consistently reflect the exploratory nature of the study (such as page 1, lines 1-3; page 2, lines 40-54).

At the same time, we respectfully note that all eight patients who were AIEC-positive at baseline were AIEC-negative at week 12 following FMT. While spontaneous fluctuation in AIEC carriage has been reported, consistent and complete clearance across all baseline-positive individuals is uncommon. Although this observation cannot establish causality in the absence of a control group, it supports the biological plausibility of an FMT-associated effect and justifies further investigation in controlled trials.

Regarding statistical reporting, we agree that consistency and rigor are essential. We have reviewed all analyses in consultation with a professional statistician (Mr. Yee-Kit Tse, now acknowledged in the manuscript). Terminology such as "trending" or "approaching significance" has been removed, and results with $p \geq 0.05$ are now uniformly described as non-significant. Multiple-testing procedures are clearly specified where applicable (page 14, lines 510-519). We believe these revisions substantially strengthen the clarity, balance, and methodological transparency of the manuscript.

2. This permeates the paper from Table 1 - comparisons of unbalanced sample sets (9 vs 21) by Student's T-test require that the samples are homoscedastic but this is often violated with sample sample numbers (eg. 9). The authors did not test for violation of this. By contrast, Welch's T-test does not assume equal variance, and is robust to unequal sample sizes.
Response: We thank the reviewer for highlighting this important methodological point. We regret that the original description lacked sufficient clarity. Upon careful review of our analysis code, we confirmed that comparisons of continuous variables in Table 1 were conducted in R using the t.test() function with default settings (var.equal = FALSE), which implements Welch's t-test. This approach does not assume equal variances and is robust to unequal sample sizes, making it appropriate for comparisons between groups of 9 and 21 participants. We have revised the Statistical Analysis section (page 14, lines 500-502) and the footnote to Table 1 to explicitly state that Welch's t-test was used for continuous variables. We appreciate the reviewer's comment, which has allowed us to improve the methodological transparency and accuracy of the manuscript.

3. Metaphlan analysis: significant results and results 'trending' to significance are

discussed but there is no discussion of the effect size. Only calculated Shannon index, without also calculating the Peilou's evenness, richness and Faith's measures. Differences between these would have enabled greater insight into the structure of the population changes that were happening.

Response: We thank the reviewer for this constructive and insightful comment. Regarding the MetaPhlAn analyses, differential abundance testing was performed using MaAsLin2. To enhance interpretability, we now explicitly report effect sizes (MaAsLin regression coefficients) in the Results section and provide the complete set of coefficients with corresponding FDR-adjusted $q$values in the source data files. In addition, we have removed language referring to results as “trending” and report non-significant findings consistently. To provide a more comprehensive assessment of alpha diversity, we have expanded our analyses beyond the Shannon index. For the fecal metagenomic dataset, Observed Richness and Pielou’s Evenness have been added (Supplementary Figure S3, related to Figure 2b). Neither metric changed significantly following FMT (Richness $p = 0.300$; Evenness $p = 0.085$). For the mucosal 16S dataset, Observed Richness, Pielou’s Evenness, and Faith’s Phylogenetic Diversity have been added (Supplementary Figure S10, related to Figure 3b). Pielou’s Evenness increased significantly following FMT ($p = 0.046$), whereas Richness ($p = 0.058$) and Faith’s PD ($p = 0.262$) did not. These additional metrics provide greater ecological context regarding community structure without materially altering the overall interpretation. Finally, we corrected a typographical error in the legend of Figure 3b, replacing “paired fecal samples” with “paired mucosal samples.”

4. How did the authors confirm that the AIEC were cleared from the samples and did not just drop below the level of detection of the method they used?

   Response: We thank the reviewer for this important and thoughtful question. We acknowledge that we cannot completely exclude the possibility that AIEC persisted at levels below the detection threshold of our methodology, as this limitation is inherent to both culture-based and sequencing-based approaches. In our study, AIEC detection was performed using direct mucosal bacterial isolation followed by a functional adhesion–invasion assay adapted from O’Brien et al. (2016; PMID: 27196580), which is widely regarded as the reference standard for identifying viable, pathogenic AIEC from mucosal samples. Post-FMT, no isolates fulfilling the established AIEC criteria were recovered. To ensure precision in terminology, we have clarified in the revised manuscript (page 10, lines 333-336) that “decolonization” refers to the absence of detectable AIEC using this validated assay, rather than definitive proof of absolute eradication. We agree that future studies incorporating complementary quantitative or molecular approaches may help further refine sensitivity and detection thresholds.

5. With respect to the inconsistent use of p-value cut-offs, the authors selectively report results that help build their story using 'trends' (e.g. "However, among recipients, 377 higher species-level engraftment tended to correlate with greater reductions in fecal

378 calprotectin (log-transformed Δcalprotectin, Spearman's test, p=0.059;" "AEcAb levels remained numerically higher in the 275 baseline AIEC-positive participants but did not reach statistical significance (p=0.076;")

Response: We agree that statistical thresholds must be applied and reported consistently. The manuscript has been revised to remove terms such as "trending" or "approaching significance." All results with $p \geq 0.05$are now reported as non-significant, without interpretative qualification (page 4, lines 107-109; page 7, lines 219-222). This ensures a consistent and objective presentation of the data throughout.

6. Is the use of a cohort with mild-to-moderate symptoms appropriate for testing clearance? This is mentioned in the discussion yet the paper over-reaches conclusions about "ability of FMT to target key pathobionts". Causal language is an issue, as study design limits the work to show assoications/correlations. Limitations should be expanded upon in the discussion.

   Response: We thank the reviewer for this important and thoughtful comment. We consider the inclusion of patients with mild-to-moderate symptoms appropriate for evaluating AIEC clearance. An actively inflamed gut may not provide a favorable "soil" for donor microbiota "seeds" to engraft and restructure the microbial ecosystem. Assessing FMT in a relatively controlled inflammatory environment therefore allows clearer evaluation of microbiological outcomes, including AIEC decolonization, without the confounding effects of severe ongoing inflammation. This rationale has been clarified in the Discussion (page 8, lines 258-278). We fully acknowledge that the study design supports associations rather than causal inference. The manuscript has been revised throughout to remove causal language, including statements suggesting an inherent "ability of FMT to target key pathobionts." The conclusions are now consistently framed as exploratory and hypothesis-generating. In addition, the Discussion has been expanded to more explicitly highlight the principal limitations namely the absence of a control group, the limited sample size, and the inclusion of patients in remission or with mild-to-moderate disease activity and to clearly state how these factors constrain causal interpretation and clinical extrapolation (pages 9-10, lines 325-341).
